# Elucidating tissue specific genes using the Benford distribution

**DOI:** 10.1186/s12864-016-2921-x

**Published:** 2016-08-09

**Authors:** Deepak Karthik, Gil Stelzer, Sivan Gershanov, Danny Baranes, Mali Salmon-Divon

**Affiliations:** Department of Molecular Biology, Ariel University, Ariel, 40700 Israel

**Keywords:** Benford law, RNA-seq, Gene expression

## Abstract

**Background:**

The RNA-seq technique is applied for the investigation of transcriptional behaviour. The reduction in sequencing costs has led to an unprecedented trove of gene expression data from diverse biological systems. Subsequently, principles from other disciplines such as the Benford law, which can be properly judged only in data-rich systems, can now be examined on this high-throughput transcriptomic information. The Benford law, states that in many count-rich datasets the distribution of the first significant digit is not uniform but rather logarithmic.

**Results:**

All tested digital gene expression datasets showed a Benford-like distribution when observing an entire gene set. This phenomenon was conserved in development and does not demonstrate tissue specificity. However, when obedience to the Benford law is calculated for individual expressed genes across thousands of cells, genes that best and least adhere to the Benford law are enriched with tissue specific or cell maintenance descriptors, respectively. Surprisingly, a positive correlation was found between the obedience a gene exhibits to the Benford law and its expression level, despite the former being calculated solely according to first digit frequency while totally ignoring the expression value itself. Nevertheless, genes with low expression that exhibit Benford behavior demonstrate tissue specific associations. These observations were extended to predict the likelihood of tissue specificity based on Benford behaviour in a supervised learning approach.

**Conclusions:**

These results demonstrate the applicability and potential predictability of the Benford law for gleaning biological insight from simple count data.

**Electronic supplementary material:**

The online version of this article (doi:10.1186/s12864-016-2921-x) contains supplementary material, which is available to authorized users.

## Background

RNA-seq is a very common application in biology to examine features of the transcriptome and global patterns of gene expression. The rapid development of massively parallel sequencing or next-generation sequencing (NGS) [1, 2] together with the reduction in sequencing cost and the maturation of analytical tools for the analysis of the data made this application a standard practice in molecular biology and medical studies. In recent years, there is a huge accumulation of RNA-seq data available in public biological databases, opening new opportunities for studying general patterns of gene expression in biological and medical systems. This copious data may now be examined using postulations that require vast information for their objective testing, such as the Benford law.

The Benford law, also known as the first digit law, contradicts intuition, by which one would assume that in any given series of numbers, the frequency of all nine digits appearing in the most significant (left-most) numeric position would be equal. The Benford law states that in naturally occurring datasets the larger digits have a lower likelihood to occur in the first digit position [3]. This law was discovered by Newcomb in 1881 who examined tables of logarithms and noticed that the first pages were used more often, as indicated by finger print stains, than later pages [4]. In 1938, Frank Benford re-discovered this phenomenon and tested it on different types of count data, including population size of different cities, rivers length, heat constants, atomic weights, electricity bills and many more [3]. Today, the Benford law is used mainly for detecting fraudulent activity in accounting and tax data reports [5, 6]. The idea of using Benford’s Law to screen data is based on the observation that regular, “naturally generated” data usually follow a logarithmic distribution, while faked data show abnormalities in the distribution [7].

Although the Benford law is known for many years, its application in biological systems was barely investigated. Benford’s law was found to be applicable to normal growth of human as well as bacterial populations [3, 8, 9]. Costas et al. found that the distribution of cell number per colony of a bacterium M. aeruginosa collected from different locations obeys the Benford law [9]. Grandison et al. [10] demonstrated that kinetic rate parameters of biological pathways follow Benford law closely. Kreuzer et al. [11] directly correlated changes in first digit distributions of EEG data with different states of anaesthesia. In the realm of genomics, it was shown that the number of ORFs for Eukaryotes follows a Benford distribution [12], Hoyle et al. [13] showed that microarray spot intensities, which are correlative to messenger RNA abundance, follow Benford distribution. Generally, first digit distribution can be used to monitor the consistency of the experimental process, and data quality [14–17].

Here we tested whether digital gene expression data (RNA-seq), generated by NGS platforms that have become the obvious choice for expression experiments, adhere to the Benford distribution. In contrast to microarray data, RNA-seq technology reflects the actual count of RNA molecules rather than inferring expression from relative spot intensity. We examined if deviation from the Benford distribution is tissue specific or influenced by changes in gene expression occurring during development. In addition, we investigated whether genes belonging to various functional categories exhibit dissimilar Benford behaviour.

## Methods

### Available RNA-seq data

Raw fastq files of a mouse liver RNA-seq sample were provided by Zahavi et al. [18]. Adapter and low quality bases were trimmed using Trim_galore [19] and reads were mapped to the mouse genome (build mm10) using TopHat2 [20]. HTSeq-count script [21] was used in order to count the reads mapping each annotated mouse gene, generating a count table. Frequency of the most significant digit was calculated as described in the “Benford analysis” section below.

RNA-seq raw gene count datasets were downloaded from the ReCount resource [22]. These include the Illumina Human BodyMap 2.0 data set [Gene Expression Omnibus accession code GSE30611] that consists of 16 human tissue types, and the transcriptome data of Drosophila Melanogaster at different developmental stages [23]. “Globally normalized” RNA expression (given in RPKM values) of human tissues from multiple donors was downloaded from the GTEx portal [24]. Single-cell gene expression was obtained from the GEO portal. In these experiments, RNA isolated from 44,808 mouse retinal cells (GSE63472) and 11,149 mouse ES cells at various differentiation time points (GSE65525) were sequenced and profiled using the Drop-seq technology [25, 26]. The raw gene count tables were obtained from GEO, and converted to counts per million (CPM) values prior to mean absolute error (MAE) calculation (see below).

### Simulations for dissecting technical parameter effect

The raw data for this analysis originated from the ABRF SEQC study which includes two sample types. The first is the Universal Human Reference RNA (740000, Agilent Technologies) and the second is the Ambion FirstChoice Human Brain Reference RNA (AM6000, Life Technologies). Both of which are well characterized standards that were used as part of the SEQC study by the US Food and Drug Administration (Seqc/Maqc-III Consortium. [27]). In contrast to the brain tissue samples, the universal human reference pools 10 human cell lines. Three paired-end 100 bp replicates were selected and downloaded (Gene Expression Omnibus accession GSE47792) for each sample type.

In order to simulate the effect of sample origin (cell lines vs tissue), sequencing length, sequencing type (paired or single-end) and sequencing depth on the Benford behaviour, the following analyses were performed: (1) Original 100 bp paired-end reads for both sample origin types (2) 100 bp single-end reads for both sample origin types, in this case only the left reads were used (3) Single-end reads that were computationally trimmed to 50 bp (4) Single-end reads that were computationally trimmed to 25 bp. Instead of using all of the original paired-end reads, we randomly chose (5) 80 % (6) 50 % and (7) 30 % of the sequences. For each simulation, adapter-trimmed (using Trim Galore [19]) raw sequences were aligned to the hg38 genome assembly (UCSC) with Tophat2 aligner version 2.0.1 [20]. HTSeq-count script [20] was used to generate counting tables describing the number of reads falling within each annotated gene. Unless specified otherwise the Bioconductor edgeR package [28] was used to calculate various expression metrics. The Benford test (see below) was applied to the following expression data: (1) raw counts (2) Counts Per Million (CPM) mapped reads values (3) Reads Per Kilobase of transcript per Million mapped reads (RPKM) (4) Gene based Transcripts Per Million (TPM) values, calculated using an in-house R script.

In total, 168 matrices were computed (four gene expression calculation methods for 42 [three replicates of seven technical parameters tested for two sample origins: tissue vs. cell line] generated datasets).

### Lists of housekeeping and tissue specific genes

A list of human housekeeping genes was obtained from Eisenberg et al. 2013 [29]. Tissue specific genes were obtained from the GeneCards database [30, 31]. Out of the 466 lung tissue specific genes, 306 which had matched gene symbols in GTEx were used in downstream analysis. A similar number of housekeeping genes were randomly chosen out of the 3701 that were downloaded. Due to the lack of available mouse housekeeping and retina-specific genes, we used the human lists after converting the human gene symbol to their mouse orthologues. A list of 296 retina-specific genes was fetched from the GeneCards database, together with their homologous mouse gene symbols. The list of ~300 human housekeeping genes used above was converted to mouse gene symbols using BioMart Ensembl tool [32].

### Benford analysis

The first digit distribution was determined for the different gene expression count datasets. The first digits distribution of the read counts were calculated, while ignoring zero values. All included datasets were compared to the Benford distribution using the R package BenfordTests [33] and in-house scripts. The mean absolute error (MAE) defined in the following formula

$$ MAE=\frac{1}{n}{\displaystyle \sum_{i=1}^n\left| Ai-Ei\right|} $$

was used in order to measure the amount of deviation from the Benford distribution, where Ai is the observed frequency of first digit i, Ei is the expected value as predicted from the Benford distribution and n equals 9.

Quantile normalized lung gene expression data (given in RPKM values) from 133 individuals originating from the GTEx database was analysed for a subset of genes belonging to either tissue-specific, housekeeping or random categories (approximately 300 genes of each). The mean absolute error (MAE) from the Benford distribution was calculated in two ways. In the individual-centric mode, the MAE was calculated for every gene category in each sample (individual) such that three MAE values were generated per individual for either a tissue specific, housekeeping or random gene set. The distribution of these values across individuals was then plotted for each gene category. In the gene-centric mode, the MAE was calculated across individuals for every single gene included in the different gene categories. The distribution of these MAE values within each category was plotted.

In the retina single-cell analysis, genes were defined as expressed if their mean CPM (counts per million mapped reads) values calculated across all cells were in the top 40 % [34]. Since genes which are not expressed inherently deviate from the Benford law, we pre-filtered for expressed genes prior to their ranking according to MAE scores. Subsequently, genes were ranked based on their MAE values and up to 300 top and bottom genes were selected. The genes with the highest and lowest MAE scores were analysed for enriched GO terms and tissues using GeneAnalytics [35]. In the analysis of genes exhibiting both low MAE score and low expression level, we selected 321 genes having mean Log_2_CPM < 5 out of the 600 genes tested above. These genes were sorted by their MAE score value, and the top and bottom genes were analyzed using GeneAnalytics. Top genes were selected as having an MAE < 0.065 (according to the MAE distribution plot of Fig. 6c in the Results section), and a similar number of genes (25) were selected from the bottom of the list (genes having the highest MAE scores). These genes were subjected to GeneAnalytics “Tissue and Cells” analysis (based on manually curated article information as well as high throughput comparisons) [35].

In the analysis of differentiating individual mouse ES cells [26], MAE scores were calculated for every expressed gene across approximately a thousand cells at different time points (0 days representing pluripotent ES cells and 7 days representing differentiating cells) following leukaemia inhibitory factor (LIF) withdrawal. Expressed genes were defined as for the retina analysis. Genes having expression level above log_2_CPM > 8 in day 0 were selected. This group of genes was divided into two subgroups. One contains all genes having an MAE score greater than 0.04, and the other contains the remaining genes. These gene lists were subjected to descriptor enrichment analysis using GeneAnalytics.

### Multidimensional scaling classification

Gene-centric MAE values calculated for every gene across lung patients, as well as the first digit frequencies calculated per gene was used as input for Multidimensional Scaling Analysis (MDS) as well as K Nearest Neighbours (KNN) test. MDS was performed using commands in the edgeR Bioconductor package [28] The 600 Lung tissue specific and housekeeping genes were divided to training and test sets, with a proportion of 70:30 respectively. A KNN classification test using standard R functions implemented in the “class” package [36] was performed with various k values (3,5,7,9). Optimal results were observed with *k* = 7.

### Statistical test

In order to determine if a numerical data could conform to the Benford law, Pearson’s Chi-squared Goodness-of-Fit test was performed (see R BenfordTests package [33] for more details). The null hypothesis is that the population’s first digits distribution conforms to Benford’s Law, hence a distribution having a *p*-value > 0.05 is considered to adhere to the Benford distribution. A comparison between distributions was done using the Mann–Whitney-*U* test.

## Results

### Benford distribution in digital expression data

**Fig. 1:**
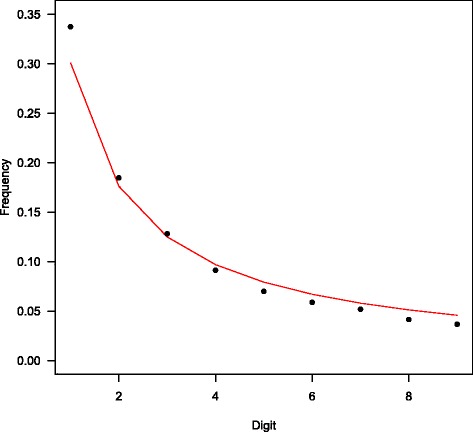
The proportional frequency of each leading digit as predicted by either the Benford distribution (*solid line*) or observed in mouse liver RNA-seq data (*black circles*)

In order to test if RNA-seq gene expression data follow Benford’s law, we used mouse liver sequencing data [18]. Calculation of the most significant digit frequency revealed that the digits of mouse liver expression data are not uniformly distributed, but rather similar to the Benford distribution (Fig. 1). Whilst Chi-squared Goodness-of-Fit test rejected the null hypothesis (*p*-value < 10^−16^) probably due to the slight deviations in the first digit frequencies, the Benford trend is clearly discernible. Digit 1 appears approximately 30 % of the time as the most significant digit, and is more frequent than other digits, which have progressively reduced frequencies.

**Fig. 2:**
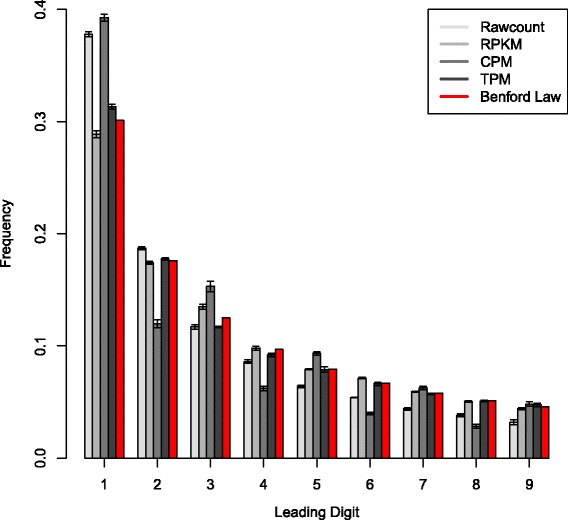
First digit frequencies of expression data, calculated for different expression metrics. Expression data was calculated based on 100 bp single-end reads of the Ambion FirstChoice Human Brain Reference RNA-seq. The mean + SD across three replicates are shown. Red bars represent the expected Benford distribution

Next we tested the effect of different RNA-seq technical parameters, such as library type, read length, coverage, sample origin (cell line vs tissue), as well as different ways to calculate gene expression (raw counts vs various normalizations) on the obedience to the Benford law (see Methods for details). Our broad simulation analyses demonstrate that the expression-based Benford pattern does not depend on read length, coverage and library type (Additional file 1: Figure S1, Additional file 2: Figure S2, Additional file 3: Figure S3, Additional file 4: Figure S4, Additional file 5: Figure S5, Additional file 6: Figure S6, Additional file 7: Figure S7, Additional file 8: Figure S8, Additional file 9: Figure S9, Additional file 10: Figure S10). Additionally, applying various normalization methods did not significantly affect the Benford trend, in which higher digits are less frequent as most significant digits (Fig. 2 for brain tissue, Additional file 11: Figure S11 for aggregated cell lines). An exception to this was observed when looking at CPM values (Fig. 2, Additional file 4: Figures S4, Additional file 9: Figure S9 and Additional file 11: Figure S11). Ignoring decimal numbers below 1, which are typical of very lowly expressed genes, restores the Benford pattern (Additional file 5: Figure S5, Additional file 10: Figure S10). Importantly, the preservation of lognormal distribution is vital for observing the Benford pattern. Removal of the log nature of the data by transforming any type of gene count into a log scale will rescind this effect (Additional file 12: Figure S12). The Benford distribution was manifested in all replicates as demonstrated by a small standard deviation (Fig. 2). Since various metric generating methods (raw counts, RPKM, TPM) exhibit the Benford pattern, they are interchangeable for testing additional Benford-related characteristics. In downstream analysis we used either raw counts or RPKM values. In analyses that ignore lowly expressed genes, the CPM values were used as well. The various expression metrics that were used in different analyses are summarized in Additional file 13: Table S1.

**Fig. 3:**
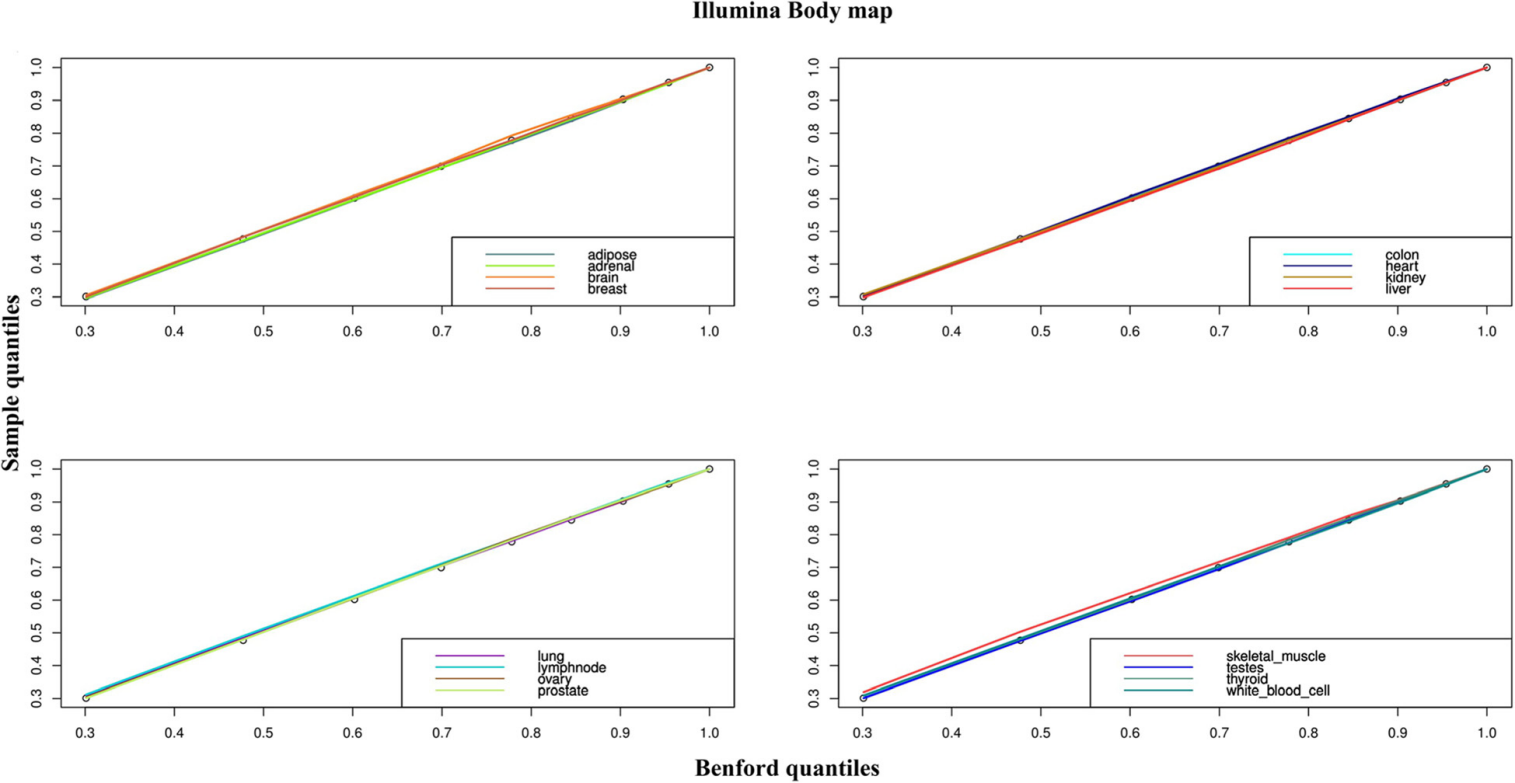
QQ-plots comparing the first digit frequency of the Illumina Human Bodymap 2.0 data to the Benford distribution. All tissue distributions are close to the bisecting line representing agreement with the Benford law

Even though the genetic makeup of all cells in the body is identical, expression levels of the general populations of genes varies between different tissues and cell types. Therefore, the observation of adherence to the Benford distribution in the liver as described above was ascertained in 16 human tissues using the Illumina BodyMap 2.0 dataset. The distribution of the first digit frequency derived from each tissue expression table was compared with the Benford distribution using the Pearson’s Chi-squared Goodness-of-Fit test, leading to a P-value larger than 0.1 for all but two tissues (brain, skeletal muscle), clearly accepting the null hypothesis that the samples adhere to the Benford distribution. This is confirmed by corresponding quantile (Q-Q) plots (Fig. 3) which indicates almost no deviation from the diagonal line, even for the two tissues that did not pass the statistical test detailed above. These results demonstrate that the compliance of gene expression data with the Benford law is a global pattern which is not tissue specific.

### Benford law adherence in gene categories

**Fig. 4:**
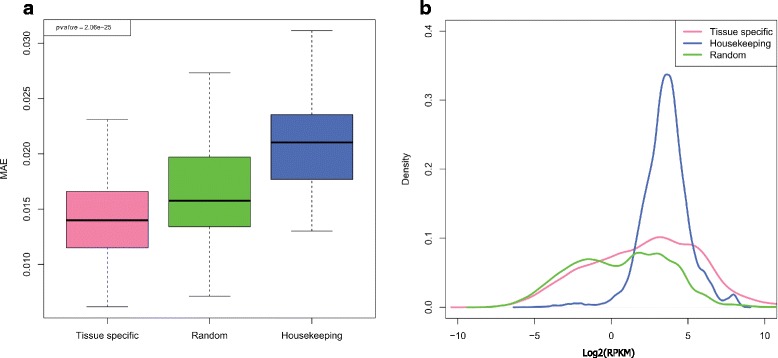
Expression deviation of different gene sets from the Benford distribution: **a** MAE (mean absolute error) distributions across 133 lung tissues for housekeeping, tissue specific and random gene sets (individual-centric mode). A one-sided Mann–Whitney *U* test was computed to compare between the distributions of tissue-specific vs. housekeeping genes, and the p values are indicated in the plot. **b** Density plots for gene expression values according to the aforementioned categories. Gene expression values from all individuals were binned

Next, we sought to test whether different gene types such as housekeeping and tissue specific genes, which are exposed to diverse transcriptional regulation, exhibit variations in their obedience to the Benford distribution. Housekeeping genes are constitutively expressed in all tissues to maintain cellular functions, but are presumed to produce the minimally essential transcripts necessary for normal cellular physiology [37]. On the other hand, tissue specific genes show an elevated expression in a particular tissue where their function is required. In order to test the agreement of these gene types with the Benford distribution, we used the RNA expression data from the GTEx portal [24]. In contrast to the Illumina body map project, which tested expression in a single sample from different tissues, the GTEx database contains tissue expression from multiple donors. This enables examination of the Benford distribution of a specific gene or a gene set across many individuals. Lung expression data was subjected to individual-centric Benford distribution deviation (MAE, see Methods) calculation for each individual and across either tissue-specific, housekeeping or random gene categories. Distribution of MAE values was highest in housekeeping genes, and lowest in the tissue specific gene set (Fig. 4a). A similar and even stronger pattern was exhibited when calculating the MAE for every gene across all individuals (gene-centric mode) thereupon plotting the distribution according to gene categories (Additional file 14: Figure S13). Additional tested tissues (brain and heart, Additional file 15: Figure S14a, b) exhibited results along the same line, indicating that this is probably a general phenomenon. When looking more closely at the expression levels of the three gene sets (Fig. 4b), we could clearly see the narrow distribution of the housekeeping genes’ expression levels compared with random and tissue-specific genes. This is in agreement with the principle that data is likely close to the Benford distribution if it is spread widely, i.e., its values span multiple orders of magnitude [38, 39].

### Benford and single-cell transcriptome

**Fig. 5:**
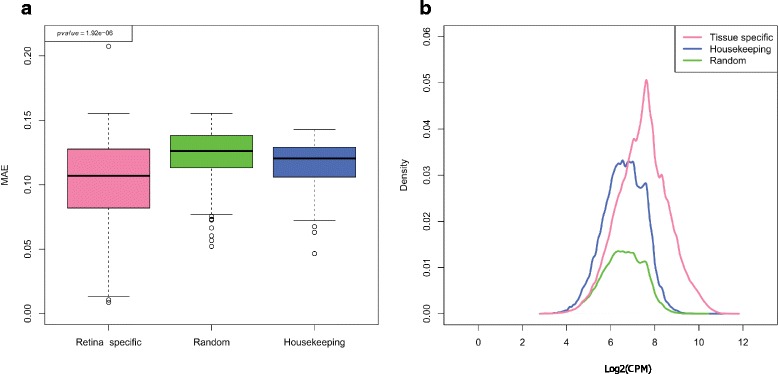
Expression deviation of different gene sets from the Benford distribution: **a** MAE (mean absolute error) distributions across ~44,000 retina cells for housekeeping, tissue specific and random gene sets. A one-sided Mann–Whitney *U* test was computed to compare between the distributions of tissue-specific vs. housekeeping genes, and the p values are indicated in the plot. **b** Density plots for gene expression values according to the aforementioned categories. Gene expression values from all individuals were binned

Recently, novel technologies enable the examination of cell-specific gene expression across a tremendous amount of single cells [25, 40, 41]. This markedly advances our capacity to understand individual cell heterogeneity within a single tissue, not possible using whole tissue RNA-seq data, such as those available for several hundreds of samples as in the GTEx database [24]. In order to test whether the deviation pattern from the Benford distribution observed for whole tissue is preserved across single cells we used RNA-seq data generated for ~44,000 mouse retinal cells [25]. The gene-centric mode MAE score for retina-specific genes, identified via the GeneCards database search engine, as well as random and housekeeping genes across all cells was calculated and the distribution of these scores is presented in Fig. 5. The pattern observed for both whole tissue as well as individual cells are in concordance (housekeeping genes having higher MAE score distribution than tissue-specific genes), albeit the differences among the various gene sets were much less pronounced in the single cell data.

**Fig. 6:**
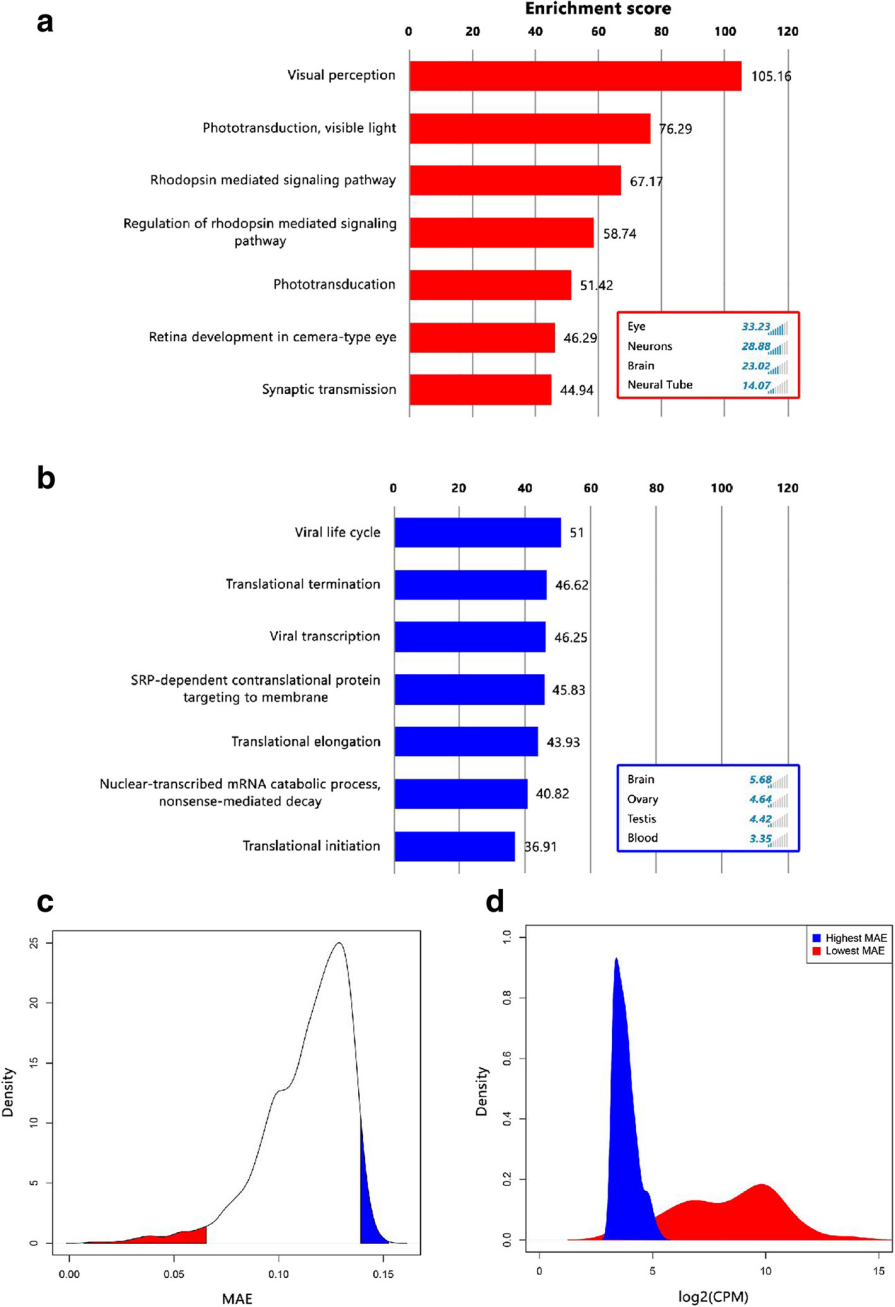
Benford analysis of single-cell retinal RNA-seq data: GeneAnalytics analysis of the extremely deviating genes from the Benford distribution. Least (**a**) and most (**b**) 300 (in each direction) deviating genes were subjected to enrichment analysis of Gene Ontologies – Biological Processes (*main panel*) and Tissues and cells (*inner panel*). **c** The distribution of MAE (mean absolute error) scores from the Benford law for all genes. Highest (*blue*) and lowest (*red*) 300 scoring genes were selected for further expression analysis and descriptor enrichment testing. **d** Expression level distribution of 300 highest (*blue*) and 300 lowest (*red*) MAE scoring genes

Next, in order to examine whether genes which tightly adhere to Benford can be biologically characterized, we calculated MAE scores for every expressed gene (~9800, see Methods) in the dataset across over 44,000 cell samples. The genes that adhere closest to Benford (lowest MAE scores) are involved in visual and eye related biological processes and pathways (Fig. 6a). The inner panel displaying the tissues that were enriched in the GeneAnalytics analysis, indicate that the selected 300 lowest-scoring genes are indeed associated with the eye and neural anatomical entities (neurons, brain and neural tube, Fig. 6a). The GeneAnalytics analysis of the highest MAE scoring genes are associated with GO terms or pathways which are involved in basic cellular maintenance such as translational and transcriptional processes and none were related to visual terms. Even the identified virally-oriented GO terms stem from gene subsets enriched for ribosomal proteins (Fig. 6b). Additionally, the tissues associated with the high MAE genes were not related to eye or neuron-like structures.

We subsequently tested the expression levels of the highest and lowest MAE scoring genes (Fig. 6c). In general, we observed a positive correlation between adherence to Benford and expression level. The lowest MAE scoring (most adhere to Benford) genes exhibit significantly augmented expression levels with a wider distribution than their highest MAE scoring counterparts (Fig. 6d).

Since gene ontology analysis tests for an enrichment rather than exclusiveness of biological terms in a list of genes, one would argue that the observation above in which Benford-adherence genes have tissue specific roles, relies on those genes in the list that are highly expressed in the tissue. In an attempt to address this issue, we tested whether the tissue specificity of genes residing on the lower tail of the expression distribution (where the blue and red curves overlap in Fig. 6d), can be distinguished only based on their adherence to Benford. We found, that 19 out of 25 (~76 %) genes with low expression levels, which adhere to the Benford law, were determined as associated with the eye tissue. These genes include ADAMTS1 which was suggested to be involved in the inhibition mechanism of retinal neovascularization [42] and connexin43 (GJA1) which is the major connexin protein of astrocytes in the mammalian retina [43, 44]. In contrast, only four out of 25 (~16 %) in the high MAE scoring counterparts have any association with the eye and revealed shared biological terms which are inherent in the normal metabolism of every tissue in the body, such as translational processes (initiation, elongation and termination), “nuclear-transcribed mRNA catabolic processes” and “cellular protein metabolic processes”.

### Benford in development

**Fig. 7:**
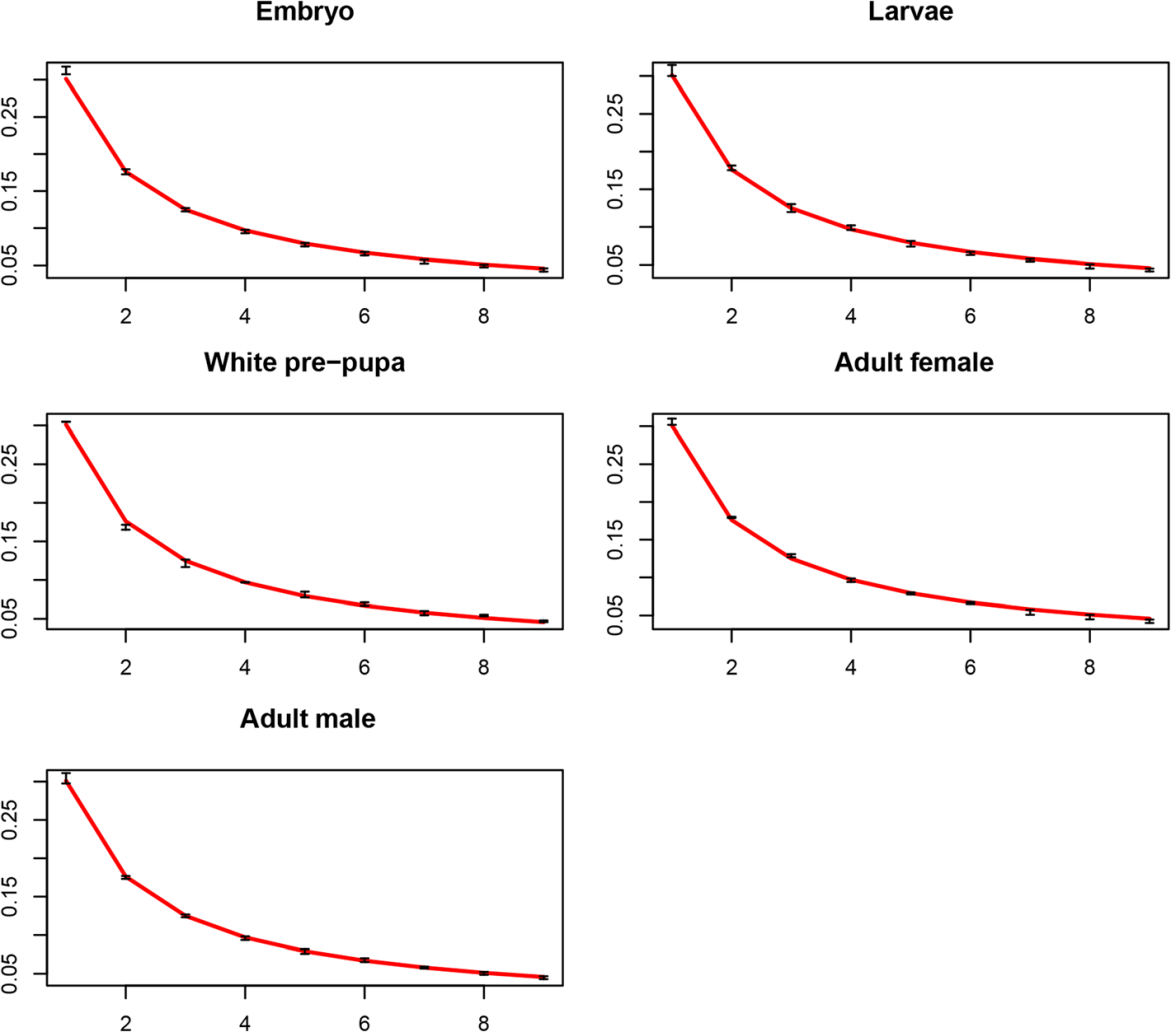
The proportional frequency of each leading digit as predicted by the Benford distribution (*solid line*) and observed in Drosophila RNA-seq data at various developmental stages. The mean + SD across replicates (2 to 12 depending on the developmental stage) was plotted

Multi-cellular organisms are able to differentially exploit their genetic information to generate morphologically and functionally specialized cell types during development. Regulation of gene expression is the major driving force of this process [45]. The diversity of expressed genes and their abundancy is highly dynamic during development, reflecting differences in requirements for basic cellular machineries in different cell types and tissues of the growing embryo. This premise was used for testing if the developmental gene expression is consistent with the Benford distribution. To this end, RNA-seq data generated for six stages during Drosophila development [23] was used as a representative developmental model system. Leading digit plots (Fig. 7) demonstrate adherence to the Benford law for global gene expression during development. The Chi-squared p-values was greater than 0.05, in at least one third of the replicates. The significant p-values observed in several replicates are probably due to small deviation of the digit 1 frequency from the expected 30.1 %, nevertheless the Benford trend is clearly evident. Focusing on genes highly expressed in adult tissues compared to all earlier developmental stages (fold-change > 16) did not change the Benford pattern in any stage (Additional file 16: Figure S15). This may be explained by the wide distribution of highly expressed adult genes in all stages, irrespective of their expression levels.

**Fig. 8:**
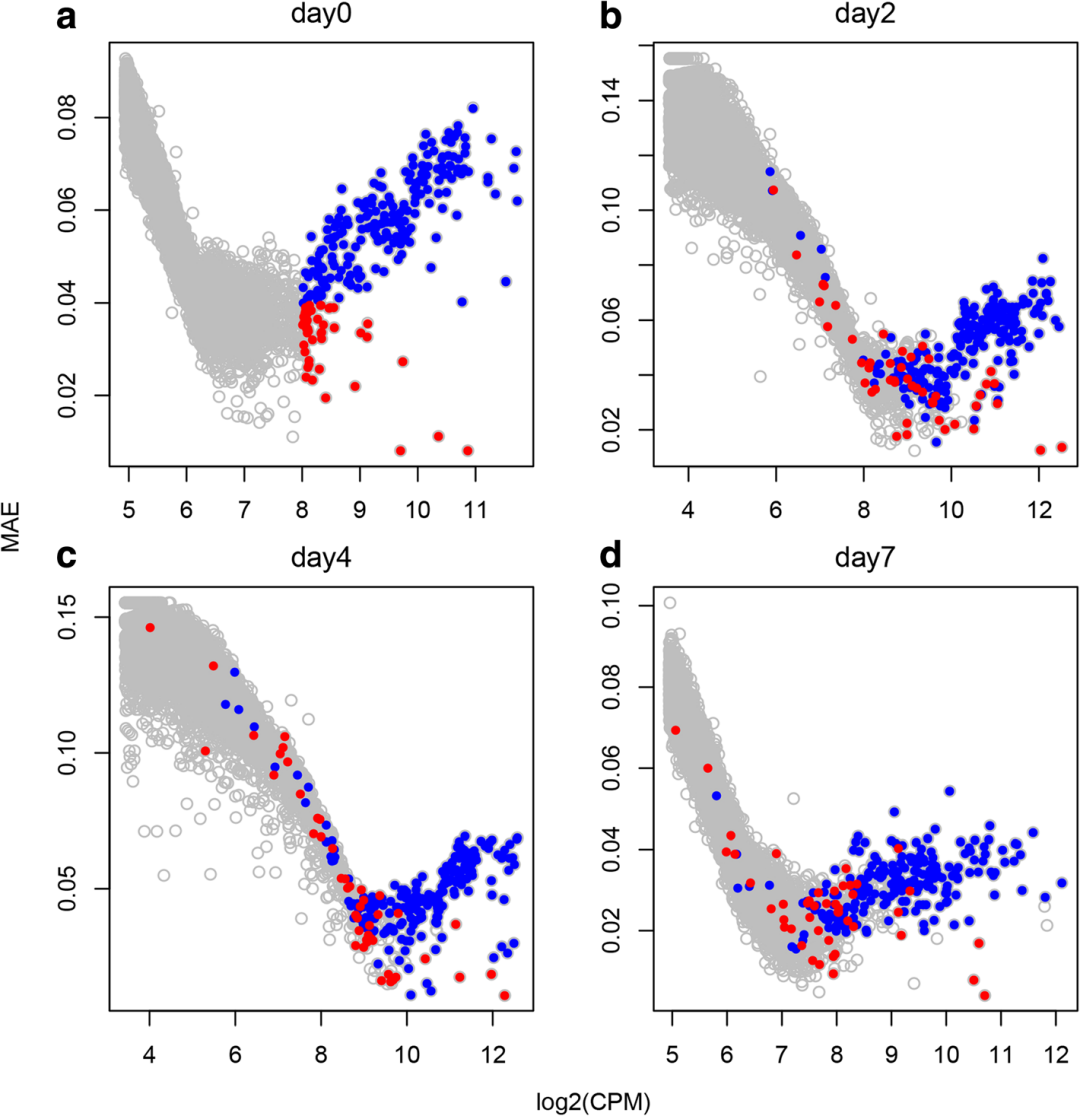
Gene expression levels plotted against MAE values, for ES cells following leukaemia inhibitory factor (LIF) withdrawal at **a** day 0, **b** day 2, **c** day 4, **d** day 7. Genes having high expression levels (log2CPM >8), and high MAE values (>0.04) in day 0 are highlighted blue. Highly expressed genes having low MAE (<0.04) in day 0 are highlighted red

In order to understand whether high resolution data could be more sensitive to changes in the Benford distribution, we performed analysis on developmental data originating from individual mouse ES cells in various differentiating stages [26]. Gene expression levels in undifferentiating ES cells (time point 0) were plotted against their MAE score (gene-centric mode calculation, Fig. 8a). A global pattern can be seen in which highly expressed genes tend to have lower MAE values (log_2_CPM between 5–8). However, this pattern does not hold for all genes. A group of genes can be clearly detected (in the right tail of the expression distribution) having very high expression levels, but higher MAE values (highlighted blue). This group is enriched with housekeeping genes having general functions such as translational processes (initiation, elongation, termination), mRNA nonsense mediated decay and structural constituent of ribosome. In contrast to this housekeeping set, we can also observe genes having high expression levels but low MAE values (highlighted red). These are enriched with cell cycle descriptors such as mitotic prophase and pathways related to G1/S checkpoint. This is in agreement with published observations whereby pluripotent ES cells are primarily in the S phase [46]. In order to test how these genes behave during development, global gene expression levels against MAE score were plotted in each time point following LIF withdrawal (day2-day7, Fig. 8b-d), and the location of the highly expressed genes (with high and low MAE score) as found in day 0 analysis was highlighted (blue and red dots, respectively). As can be seen, the housekeeping group of genes (blue) tend to keep their localized position in the plot, meaning they have high expression level and high MAE score also in advanced developmental stages which is in line with their housekeeping nature. However, day 0 low-MAE highly expressed genes lose their localized position, and are now more variable in terms of expression and MAE level.

### Benford predicting power

**Fig. 9:**
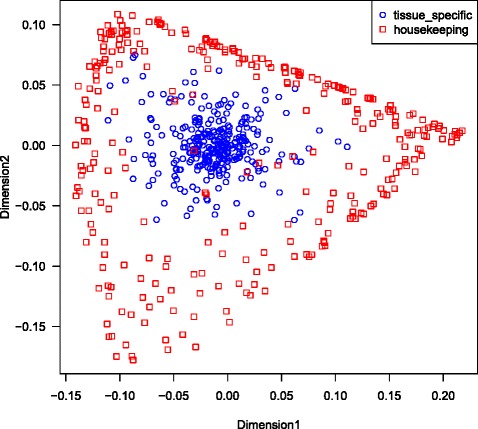
Multidimensional scaling analysis based on first digit distribution and MAE score values calculated for each gene in the GTEx lung dataset. Red squares represent housekeeping genes, while blue circles represent tissue-specific genes

As demonstrated above, tissue specific genes adhere more to the Benford law than housekeeping genes. In order to test if tissue-specific genes can be clustered together only based on their Benford behaviour, we used the first digit distribution and MAE score values of each gene in the GTEx lung dataset, as input for multidimensional scaling analysis. While housekeeping genes (Fig. 9 red squares) are highly distributed in space, tissue specific genes have a unique pattern, and are clustered together (blue circles). Next, K nearest neighbours test was performed in order to investigate the feasibility of the Benford law to predict the tissue specific tendency of a gene. The list of tissue specific and housekeeping genes was divided into training (402 genes) and test (204 genes) sets. The results of the KNN test are presented in Table 1. These results lead to a sensitivity of 0.96 while preserving high specificity of 0.95, illustrating the power of the Benford test to predict tissue specificity.

**Table 1:** KNN test investigating the predictive power of the Benford law

K nearest neighbors test (*K* = 7)		Predicted	
		Housekeeping	Tissue-specific
Actual	Housekeeping	95	5
	Tissue-specific	4	100

## Discussion

Most of the scientific literature regarding the Benford law deals mainly with its uses in the financial field, for example its application in fraud financial report detection. In life sciences, however, there is scant information regarding the uses of Benford law in biological data systems, and even less information on genomics applications. High throughput technologies provide thousands of measurements from a single biological sample, which present a tremendous source of count data against which to test Benford's law. These include gene expression counts across many individuals, and more recently, single cell measurements, which allow testing of heterogeneity in the nature of gene expression across single cells. Here we report that digital gene expression follows Benford distribution in a wide range of biological tissues and developmental conditions. Although read length and coverage highly influence the ability to quantify differential gene expression [47, 48] they have a negligible impact on the Benford behaviour of gene expression data.

In general, numerical data which follows the Benford distribution, usually have a logarithmic nature [4]. This is, therefore, the underlying explanation why digital gene expression data, which is lognormally distributed, observes the Benford law [49, 50]. This rationale may also interpret the suggestion of Hoyle et al. [13] in which gene expression adherence to the Benford law is not species specific. Indeed, our findings that gene expression data, originating from either mouse (Fig. 1), human (Fig. 3) or drosophila (Fig. 7) species follow the Benford distribution; indicate that this principle is conserved across metazoans, and may probably be extended to additional clades in the tree of life as long as the logarithmic nature of their expression data is preserved. Although the lognormal distribution of expression levels reflects true biological variability and is not an artefact of the technology [51], we still cannot rule out that the PCR exponential amplification, performed during library preparation, does not contribute to the Benford behaviour of gene expression. Therefore, the Benford distribution could be tested on PCR-free expression data such as those generated by the Nanostring technology, once these are performed on a whole genome-scale.

In order to investigate whether biological insight could be gleaned through examination of first digit frequencies, we explored these distributions in different gene sets having unique characteristics, such as tissue specific and housekeeping genes rather than scrutinizing the whole gene list. As previously described [52], tissue specific genes are expressed in fewer conditions than housekeeping. However, looking at a single condition, one tissue sample for example, the dynamic range of expression for genes, which were previously determined as tissue specific, was much wider than that observed for housekeeping genes. Our finding that housekeeping genes violate Benford's law, compared with tissue specific genes, is a reflection of their narrow expression distribution. Repeating this analysis across 133 samples of the same tissue produced the same distribution. This process was also repeated in an additional two GTEx-derived whole-tissue homogenates as well as retina single-cell data, exhibiting similar results.

The observed restricted expression range of housekeeping genes can be explained by the fact that housekeeping genes do not map to random locations throughout the human genome, but instead resolve to clusters [53, 54]. This may subject the clustered genes to the same transcriptional control, leading to a narrow expression range. In contrast to housekeeping genes, tissue-specific genes exhibit a wide expression dynamic range which explains their Benford behaviour. This wide range is surprising in itself since one would expect tissue specific genes, which are defined as genes whose expression is vital to the normal metabolism of the tissue, to demonstrate a narrow distribution of high expression level. Our data suggest that tissue specificity and expression distribution (within a single condition/tissue) are orthogonal characteristics of genes.

It is recommended to analyse large datasets (>1000) in order to discern Benford tendencies [55]. This requirement can be easily met by observing the expression of many genes in a single tissue RNA sample. However, in order to analyse the Benford distribution of a single gene, the recommended experiment sample size should reach a thousand samples, which for the most prevalent RNA-seq experiments, is not practical.

The advantage of high throughput single-cell sequencing technologies is the possibility to dissect the expression of a single gene across a vast amount of samples. We harnessed the availability of two highly parallel single-cell expression profiling datasets available for mouse retina and ES cells, to rank individual genes in accordance with their closeness to the expected Benford distribution. Once this rank was available we could inspect whether it is biologically meaningful. It is unexpected that genes that were selected based only on their Benford distribution property, while completely ignoring their expression value, will share unique biological characteristics. Surprisingly, we found that genes exhibiting the Benford pattern are more likely to have a functional role within the tissue in question, and are likely to be highly expressed. Furthermore, we observed that Benford-adherent genes with low expression levels tend to have tissue oriented functionality rather than basic maintenance functions (translation and transcription processes) which characterise their Benford-divergent counterparts. Therefore, genes that were overlooked for roles in tissue functionality, due to their lower expression level, should now be revaluated for this capacity based on their Benford behaviour. This could be achieved by possibly overexpressing or completely eradicating their expression, thereupon examining the resulting phenotype in the tissue or cell line in question, where they are predicted to have specific roles.

Two approaches were taken in this study in order to test the capacity of the Benford law to predict tissue specificity. The first is by testing gene ontology enrichment of genes that were selected based on their MAE score only, without assuming anything about their nature. When we used this approach on thousands of retina single cell data, we indeed found that genes which adhere to the Benford law tend to have tissue specific roles. This phenomenon could not be observed in GTEx tissue expression levels probably due to the relatively low number of samples which are optimal for Benford analysis. Once additional high-throughput single cell data will be available, this observation could be verified in other tissues as well. The other approach uses an apriori characterised tissue specific and housekeeping gene sets, thereupon testing the structure of these datasets by visualizing the relative distance of the observations. Next, supervised machine learning quantified the feasibility of the Benford law to predict the tissue specific tendency of an unknown gene. The later was successfully applied to GTEx data despite its relatively small number of samples (133 in the lung tissue dataset).

## Conclusions

The applicability of the Benford distribution in biological datasets has not been fully realized as of yet. To the best of our knowledge, there are no previous reports in the literature showing that RNA-seq digital expression data follow the Benford distribution. Furthermore, this paper introduces the novelty of relating adherence to the Benford law within gene sets with unique characteristics, such as tissue specificity. Importantly, we demonstrated the application of Benford adherence for testing the likelihood of genes to have a general housekeeping vs. having a unique role in the examined tissue. To summarize, despite its simplicity, adherence to the Benford law is an elegant and robust means to classify genes while totally ignoring their expression level and any other gene characteristic.

## Abbreviations

CPM, counts per million mapped reads; MAE, mean absolute error; NGS, next generation sequencing

## Additional files

Additional file 1: Figure S1. The effect of different technical parameters on the Benford pattern as calculated based on brain-derived gene expression data described as raw counts. If not mentioned otherwise read length was 100 bp and all reads were used in the analysis. Truncated reads (25 and 50 bp) and lower coverage (30, 50 and 80 % out of the total reads) appear in plot titles. The red line indicates the expected Benford distribution, symbol-marked lines are the distribution observed for three replicates. (PDF 10 kb) Additional file 2: Figure S2. The effect of different technical parameters on the Benford pattern as calculated based on brain-derived gene expression data described as RPKM values. If not mentioned otherwise read length was 100 bp and all reads were used in the analysis. Truncated reads (25 and 50 bp) and lower coverage (30, 50 and 80 % out of the total reads) appear in plot titles. The red line indicates the expected Benford distribution, symbol-marked lines are the distribution observed for three replicates. (PDF 10 kb) Additional file 3: Figure S3. The effect of different technical parameters on the Benford pattern as calculated based on brain-derived gene expression data described as TPM. If not mentioned otherwise read length was 100 bp and all reads were used in the analysis. Truncated reads (25 and 50 bp) and lower coverage (30, 50 and 80 % out of the total reads) appear in plot titles. The red line indicates the expected Benford distribution, symbol-marked lines are the distribution observed for three replicates. (PDF 10 kb) Additional file 4: Figure S4. The effect of different technical parameters on the Benford pattern as calculated based on brain-derived gene expression data described as CPM. If not mentioned otherwise read length was 100 bp and all reads were used in the analysis. Truncated reads (25 and 50 bp) and lower coverage (30, 50 and 80 % out of the total reads) appear in plot titles. The red line indicates the expected Benford distribution, symbol-marked lines are the distribution observed for three replicates. (PDF 814 kb) Additional file 5: Figure S5. The effect of different technical parameters on the Benford pattern as calculated based on brain-derived gene expression data described as CPM values, ignoring very low expressed genes (CPM < 1). If not mentioned otherwise read length was 100 bp and all reads were used in the analysis. Truncated reads (25 and 50 bp) and lower coverage (30, 50 and 80 % out of the total reads) appear in plot titles. The red line indicates the expected Benford distribution, symbol-marked lines are the distribution observed for three replicates. (PDF 18 kb) Additional file 6: Figure S6. The effect of different technical parameters on the Benford pattern as calculated based on cell line-derived gene expression data described as raw counts. If not mentioned otherwise read length was 100 bp and all reads were used in the analysis. Truncated reads (25 and 50 bp) and lower coverage (30, 50 and 80 % out of the total reads) appear in plot titles. The red line indicates the expected Benford distribution, symbol-marked lines are the distribution observed for three replicates. (PDF 17 kb) Additional file 7: Figure S7. The effect of different technical parameters on the Benford pattern as calculated based on cell line-derived gene expression data described as RPKM values. If not mentioned otherwise read length was 100 bp and all reads were used in the analysis. Truncated reads (25 and 50 bp) and lower coverage (30, 50 and 80 % out of the total reads) appear in plot titles. The red line indicates the expected Benford distribution, symbol-marked lines are the distribution observed for three replicates. (PDF 11 kb) Additional file 8: Figure S8. The effect of different technical parameters on the Benford pattern as calculated based on cell line-derived gene expression data described as TPM. If not mentioned otherwise read length was 100 bp and all reads were used in the analysis. Truncated reads (25 and 50 bp) and lower coverage (30, 50 and 80 % out of the total reads) appear in plot titles. The red line indicates the expected Benford distribution, symbol-marked lines are the distribution observed for three replicates. (PDF 11 kb) Additional file 9: Figure S9. The effect of different technical parameters on the Benford pattern as calculated based on cell line-derived gene expression data described as CPM. If not mentioned otherwise read length was 100 bp and all reads were used in the analysis. Truncated reads (25 and 50 bp) and lower coverage (30, 50 and 80 % out of the total reads) appear in plot titles. The red line indicates the expected Benford distribution, symbol-marked lines are the distribution observed for three replicates. (PDF 11 kb) Additional file 10: Figure S10. The effect of different technical parameters on the Benford pattern as calculated based on cell line-derived gene expression data described as CPM values, ignoring very low expressed genes (CPM < 1). If not mentioned otherwise read length was 100 bp and all reads were used in the analysis. Truncated reads (25 and 50 bp) and lower coverage (30, 50 and 80 % out of the total reads) appear in plot titles. The red line indicates the expected Benford distribution, symbol-marked lines are the distribution observed for three replicates. (PDF 11 kb) Additional file 11: Figure S11. First digit frequencies of expression data, calculated for different expression metrics. Expression data was calculated based on 100 bp single-end reads of the Universal Human Reference RNA-seq. The mean + SD across three replicates are shown. Black bars represent the expected Benford distribution. (PDF 11 kb) Additional file 12: Figure S12. First digit distributions of the expression counts for a sample dataset (100 bp single-end reads of the universal human reference RNA-seq). First digit frequencies were calculated based on counts per million mapped reads (CPM) for all genes having (a) CPM > 0 (b) CPM > 1 (c) First digit frequencies were calculated based on log 2 of the CPM counts for all genes having CPM > 1. Red lines represent the Benford first digit frequencies together with confidence intervals. Black pluses represent the observed frequencies. Observed relative frequencies and p values are summarized below the plot (see the signifd.analysis command in the BenfordTests package for more details on the calculations). (PDF 1805 kb) Additional file 13: Table S1. The various expression metrics that were used in different analyses. (PDF 112 kb) Additional file 14: Figure S13. Expression deviation of different gene sets from the Benford distribution. The MAE (mean absolute error) was calculated across 133 lung tissues for every gene included in the housekeeping, tissue specific and random gene sets (gene-centric mode). A one-sided Mann–Whitney *U* test was computed to compare between tissue-specific and housekeeping distributions, and the p values are indicated in the plot. (PDF 5 kb) Additional file 15: Figure S14 Expression deviation of different gene sets from the Benford distribution. The MAE (mean absolute error) distribution was calculated across (a) 357 brain tissues and (b) 133 heart tissues, for every gene included in the housekeeping, tissue specific and random gene sets (gene-centric mode). A one-sided Mann–Whitney *U* test was computed to compare between tissue-specific and housekeeping distributions, and the p values are indicated in the plot. (PDF 707 kb) Additional file 16: Figure S15. The proportional frequency of each leading digit as predicted by the Benford distribution (solid line) and observed in Drosophila RNA-seq data at various developmental stages, as calculated for ~700 genes highly expressed in Adult stage compared with other stages (fold change > 16). The mean + SD across replicates (2 to 12 depending on the developmental stage) was plotted. (PDF 410 kb)
