# Supplementary figures and images for: Elucidating tissue specific genes using the Benford distribution
Source: BMC Genomics. 2016 Aug 9;17:595. doi: 10.1186/s12864-016-2921-x (PMC4979126; doi:10.1186/s12864-016-2921-x)

# Raw counts

## Paired end reads

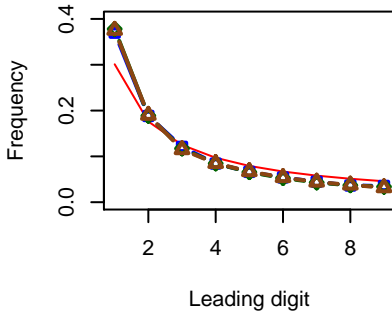

## Single end reads(SE)

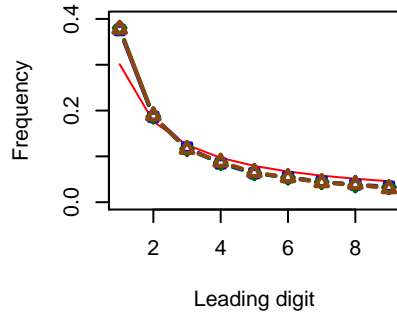

## 25bp(SE)

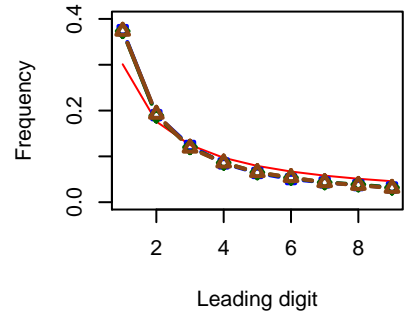

## 50bp(SE)

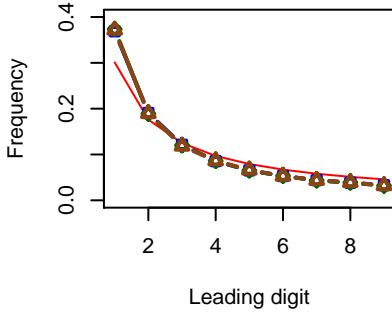

## 30%(SE)

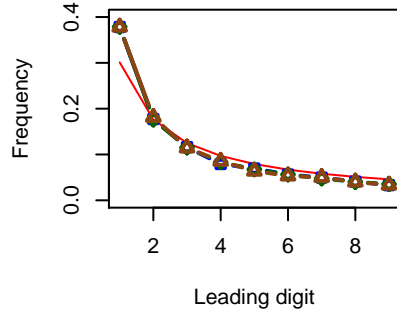

## 50%(SE)

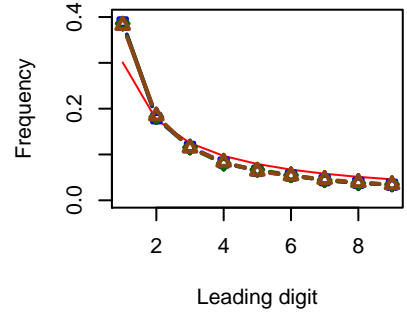

## 80%(SE)

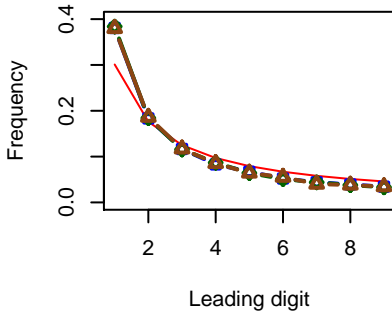

Supplement: Additional file 1: Figure S1. — The effect of different technical parameters on the Benford pattern as calculated based on brain-derived gene expression data described as raw counts. If not mentioned otherwise read length was 100 bp and all reads were used in the analysis. Truncated reads (25 and 50 bp) and lower coverage (30, 50 and 80 % out of the total reads) appear in plot titles. The red line indicates the expected Benford distribution, symbol-marked lines are the distribution observed for three replicates. (PDF 10 kb) [file 12864_2016_2921_MOESM1_ESM.pdf]

# RPKM

## Paired end reads

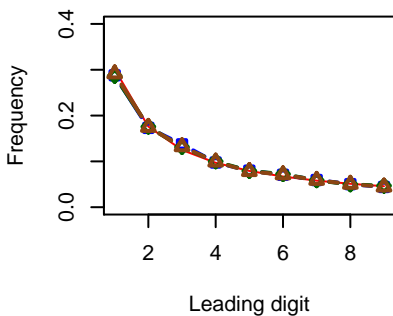

## Single end reads(SE)

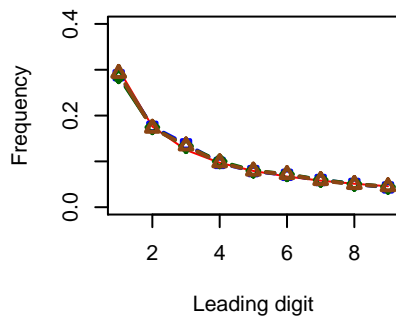

## 25bp(SE)

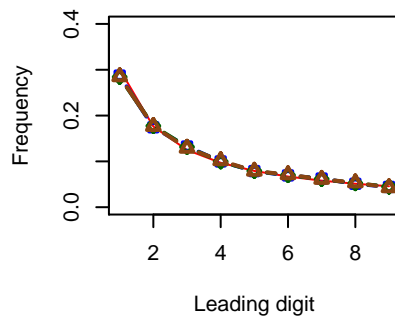

## 50bp(SE)

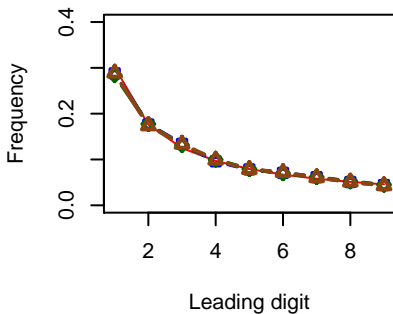

## 30%(SE)

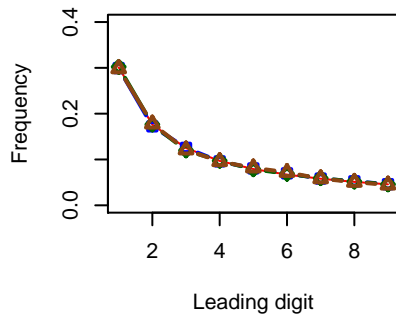

## 50%(SE)

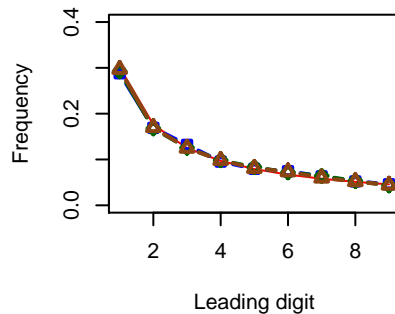

## 80%(SE)

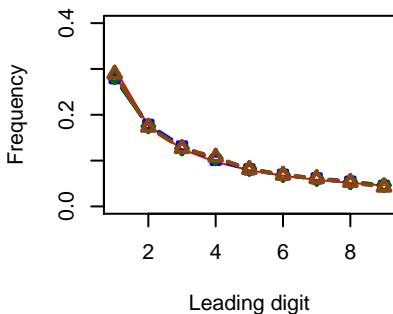

Supplement: Additional file 2: Figure S2. — The effect of different technical parameters on the Benford pattern as calculated based on brain-derived gene expression data described as RPKM values. If not mentioned otherwise read length was 100 bp and all reads were used in the analysis. Truncated reads (25 and 50 bp) and lower coverage (30, 50 and 80 % out of the total reads) appear in plot titles. The red line indicates the expected Benford distribution, symbol-marked lines are the distribution observed for three replicates. (PDF 10 kb) [file 12864_2016_2921_MOESM2_ESM.pdf]

# TPM

## Paired end reads

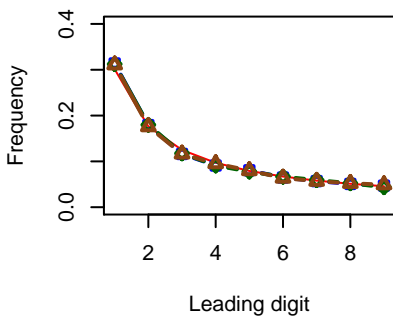

## Single end reads(SE)

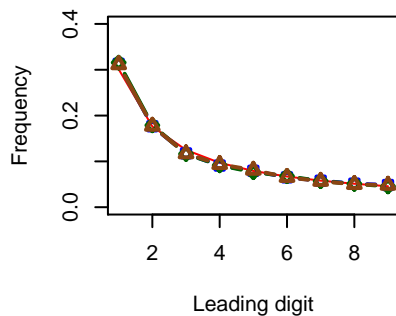

## 25bp(SE)

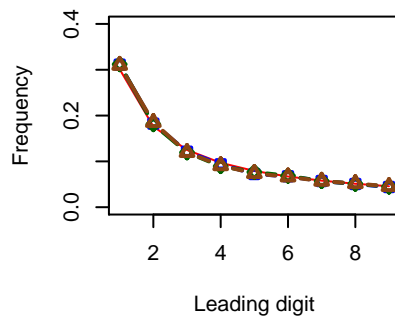

## 50bp(SE)

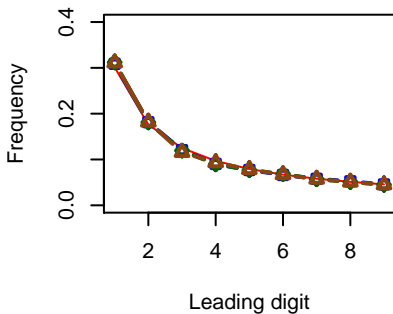

## 30%(SE)

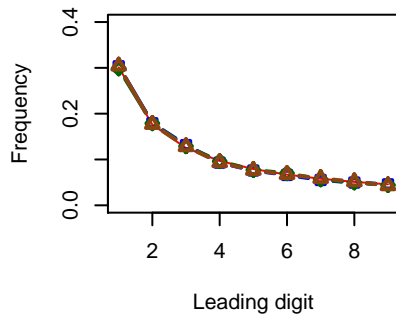

## 50%(SE)

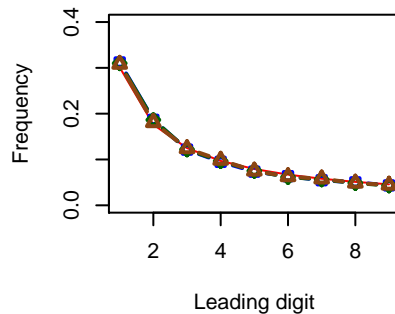

## 80%(SE)

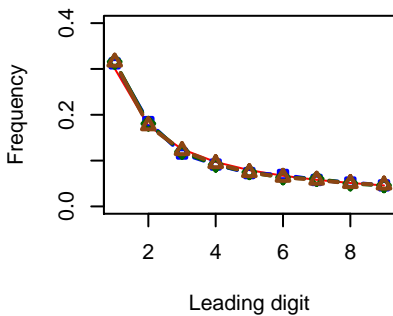

Supplement: Additional file 3: Figure S3. — The effect of different technical parameters on the Benford pattern as calculated based on brain-derived gene expression data described as TPM. If not mentioned otherwise read length was 100 bp and all reads were used in the analysis. Truncated reads (25 and 50 bp) and lower coverage (30, 50 and 80 % out of the total reads) appear in plot titles. The red line indicates the expected Benford distribution, symbol-marked lines are the distribution observed for three replicates. (PDF 10 kb) [file 12864_2016_2921_MOESM3_ESM.pdf]

# CPM

## Paired end reads

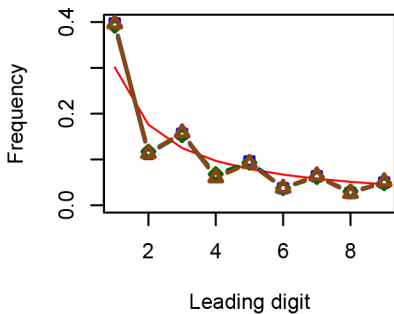

## Single end reads(SE)

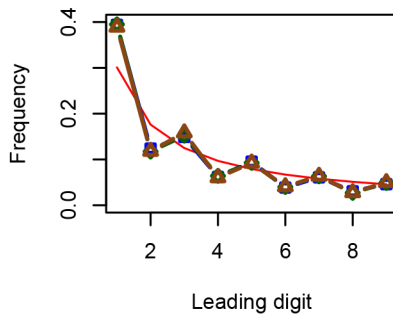

## 25bp(SE)

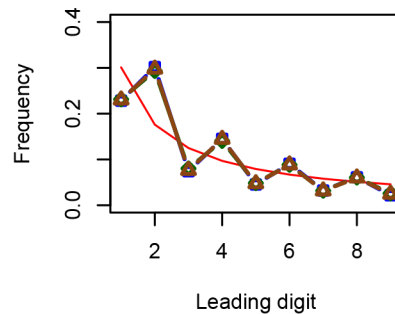

## 50bp(SE)

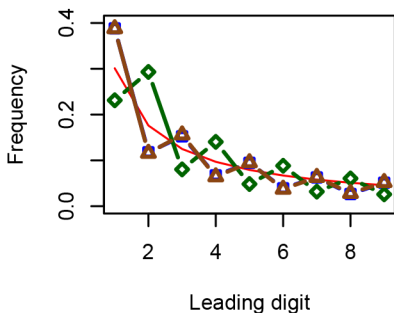

## 30%(SE)

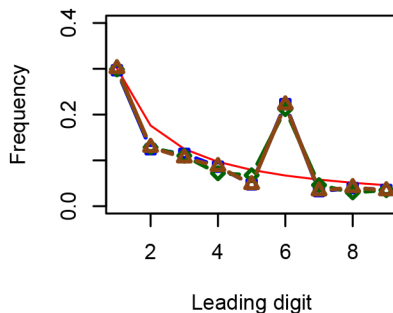

## 50%(SE)

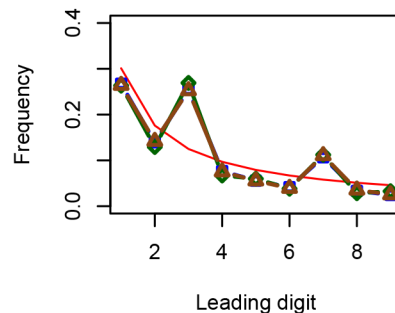

## 80%(SE)

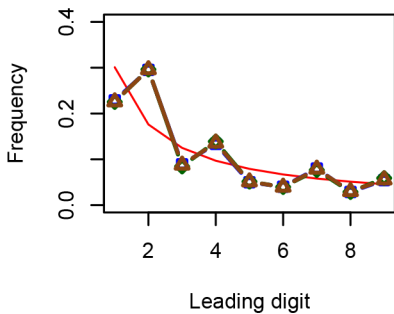

Supplement: Additional file 4: Figure S4. — The effect of different technical parameters on the Benford pattern as calculated based on brain-derived gene expression data described as CPM. If not mentioned otherwise read length was 100 bp and all reads were used in the analysis. Truncated reads (25 and 50 bp) and lower coverage (30, 50 and 80 % out of the total reads) appear in plot titles. The red line indicates the expected Benford distribution, symbol-marked lines are the distribution observed for three replicates. (PDF 814 kb) [file 12864_2016_2921_MOESM4_ESM.pdf]

# CPM

## Paired end reads

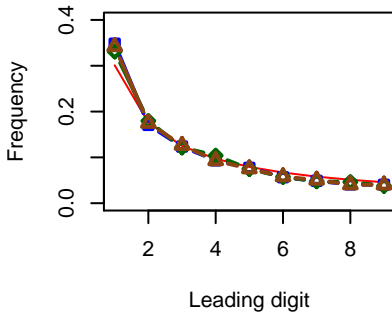

## Single end reads(SE)

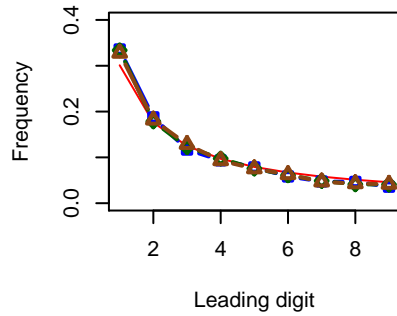

## 25bp(SE)

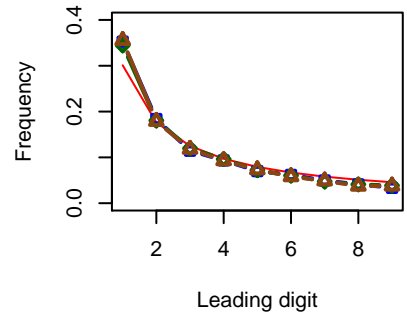

## 50bp(SE)

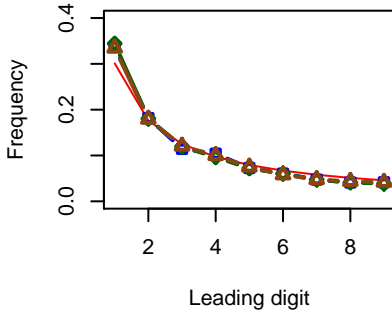

## 30%(SE)

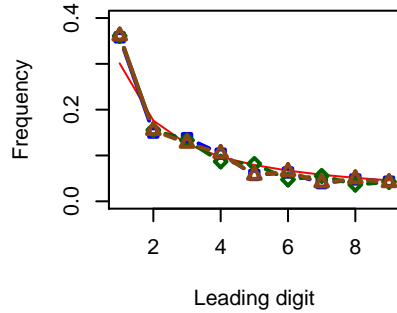

## 50%(SE)

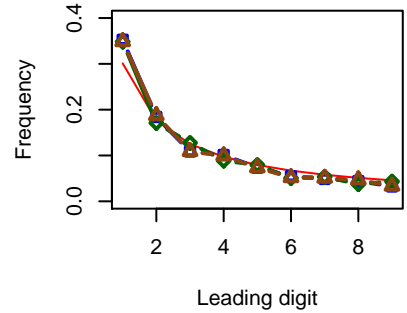

## 80%(SE)

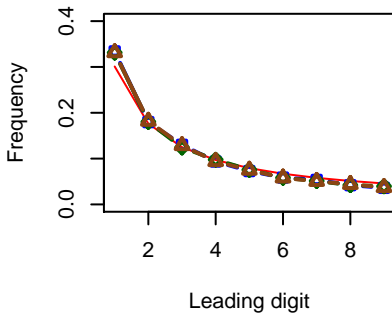

Supplement: Additional file 5: Figure S5. — The effect of different technical parameters on the Benford pattern as calculated based on brain-derived gene expression data described as CPM values, ignoring very low expressed genes (CPM < 1). If not mentioned otherwise read length was 100 bp and all reads were used in the analysis. Truncated reads (25 and 50 bp) and lower coverage (30, 50 and 80 % out of the total reads) appear in plot titles. The red line indicates the expected Benford distribution, symbol-marked lines are the distribution observed for three replicates. (PDF 18 kb) [file 12864_2016_2921_MOESM5_ESM.pdf]

# Raw counts

## Paired end reads

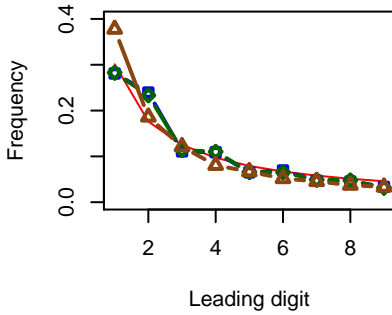

## Single end reads(SE)

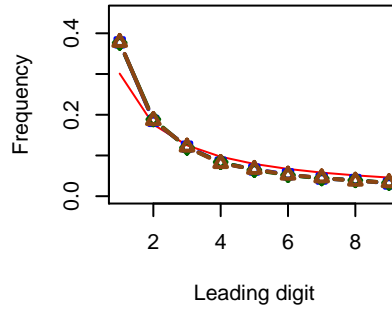

## 25bp(SE)

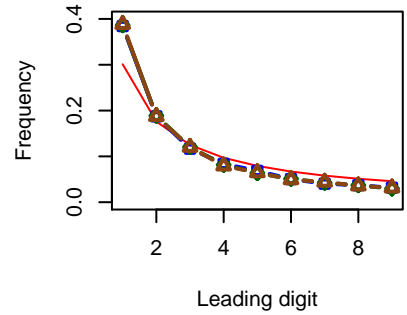

## 50bp(SE)

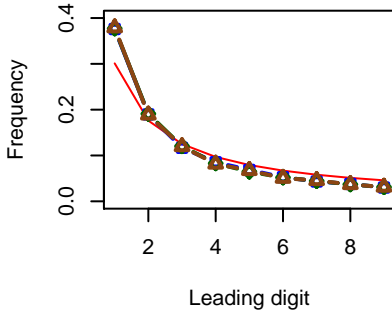

## 30%(SE)

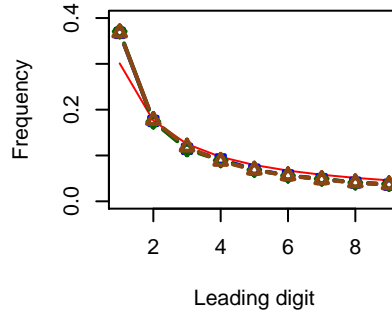

## 50%(SE)

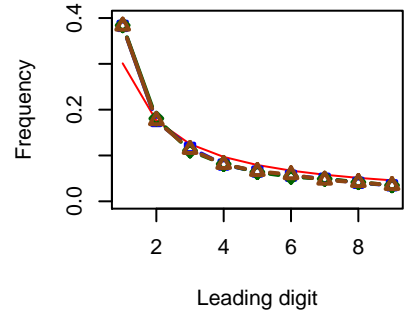

## 80%(SE)

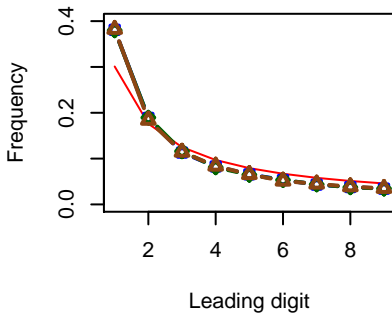

Supplement: Additional file 6: Figure S6. — The effect of different technical parameters on the Benford pattern as calculated based on cell line-derived gene expression data described as raw counts. If not mentioned otherwise read length was 100 bp and all reads were used in the analysis. Truncated reads (25 and 50 bp) and lower coverage (30, 50 and 80 % out of the total reads) appear in plot titles. The red line indicates the expected Benford distribution, symbol-marked lines are the distribution observed for three replicates. (PDF 17 kb) [file 12864_2016_2921_MOESM6_ESM.pdf]

# RPKM

## Paired end reads

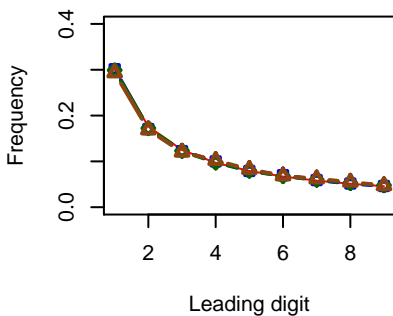

## Single end reads(SE)

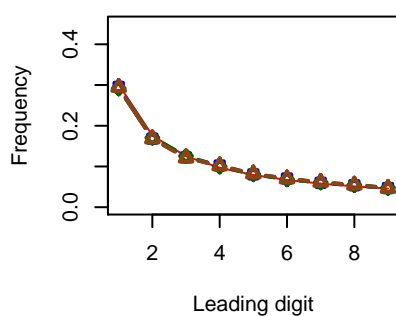

## 25bp(SE)

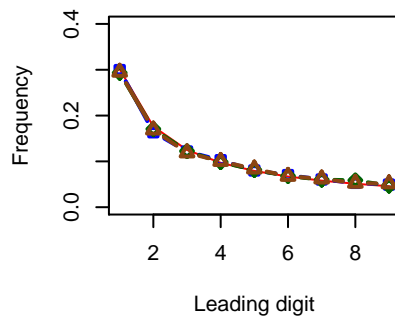

## 50bp(SE)

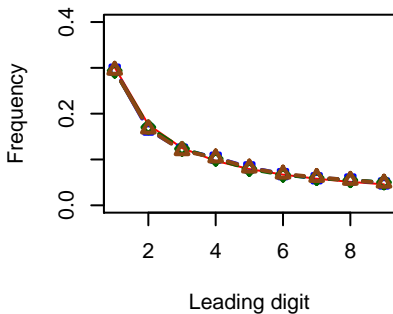

## 30%(SE)

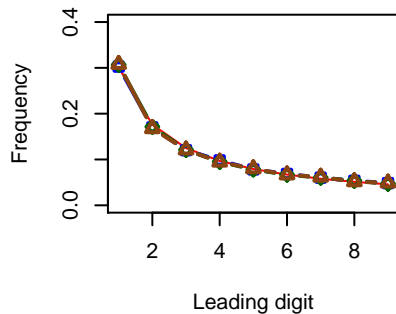

## 50%(SE)

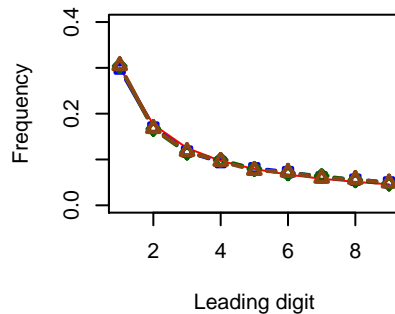

## 80%(SE)

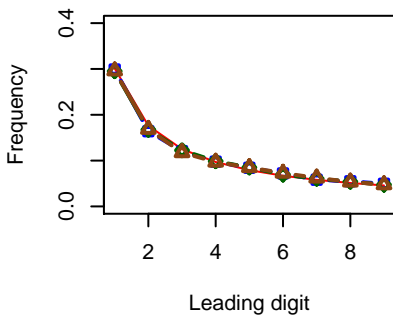

Supplement: Additional file 7: Figure S7. — The effect of different technical parameters on the Benford pattern as calculated based on cell line-derived gene expression data described as RPKM values. If not mentioned otherwise read length was 100 bp and all reads were used in the analysis. Truncated reads (25 and 50 bp) and lower coverage (30, 50 and 80 % out of the total reads) appear in plot titles. The red line indicates the expected Benford distribution, symbol-marked lines are the distribution observed for three replicates. (PDF 11 kb) [file 12864_2016_2921_MOESM7_ESM.pdf]

# TPM

## Paired end reads

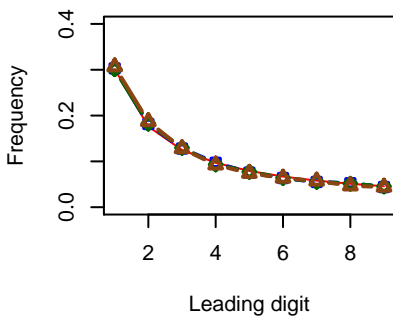

## Single end reads(SE)

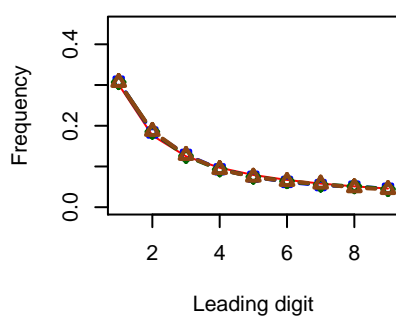

## 25bp(SE)

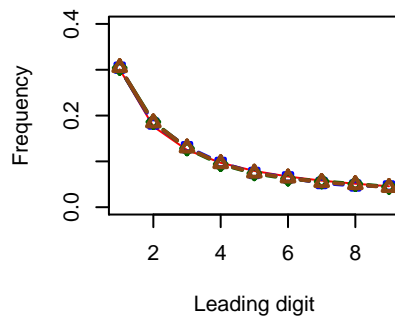

## 50bp(SE)

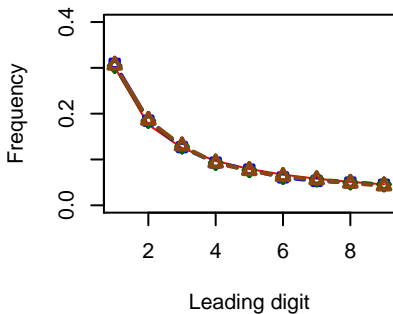

## 30%(SE)

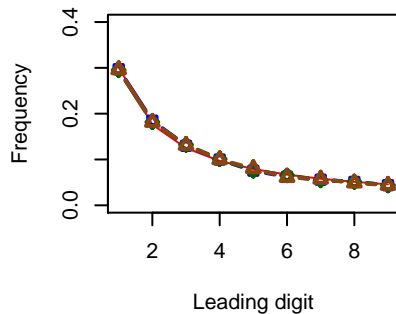

## 50%(SE)

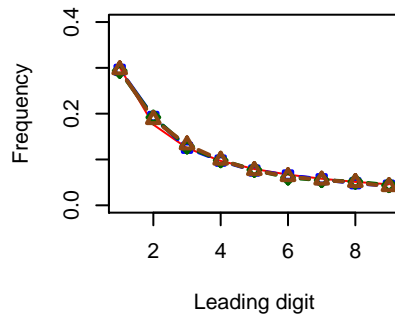

## 80%(SE)

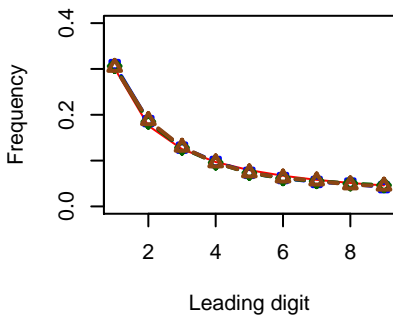

Supplement: Additional file 8: Figure S8. — The effect of different technical parameters on the Benford pattern as calculated based on cell line-derived gene expression data described as TPM. If not mentioned otherwise read length was 100 bp and all reads were used in the analysis. Truncated reads (25 and 50 bp) and lower coverage (30, 50 and 80 % out of the total reads) appear in plot titles. The red line indicates the expected Benford distribution, symbol-marked lines are the distribution observed for three replicates. (PDF 11 kb) [file 12864_2016_2921_MOESM8_ESM.pdf]

# CPM

## Paired end reads

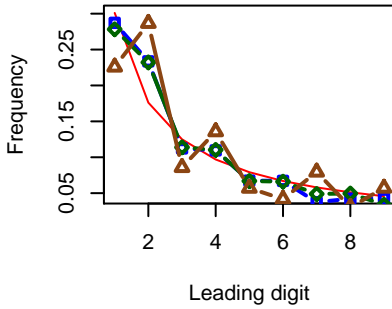

## Single end reads(SE)

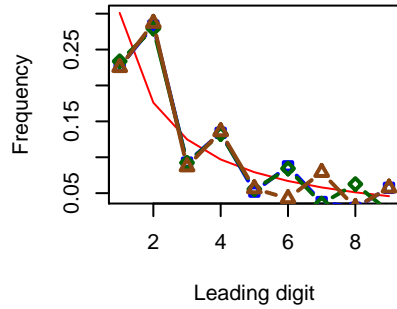

## 25bp(SE)

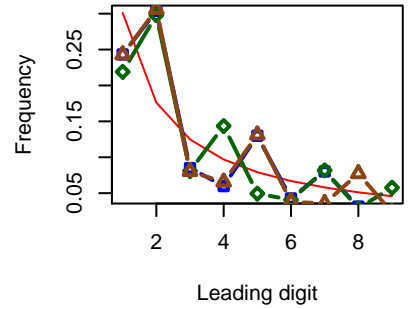

## 50bp(SE)

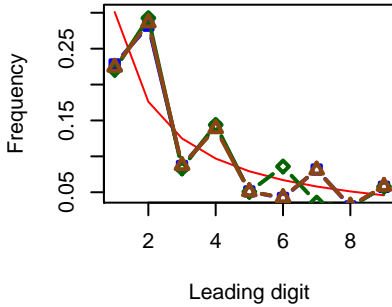

## 30%(SE)

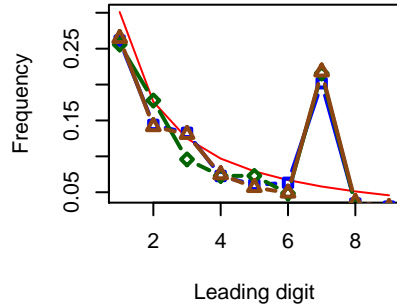

## 50%(SE)

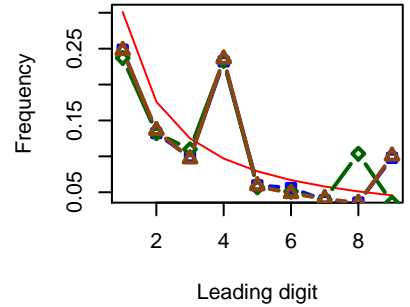

## 80%(SE)

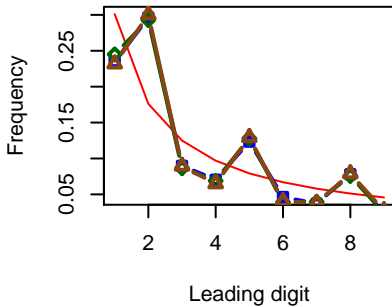

Supplement: Additional file 9: Figure S9. — The effect of different technical parameters on the Benford pattern as calculated based on cell line-derived gene expression data described as CPM. If not mentioned otherwise read length was 100 bp and all reads were used in the analysis. Truncated reads (25 and 50 bp) and lower coverage (30, 50 and 80 % out of the total reads) appear in plot titles. The red line indicates the expected Benford distribution, symbol-marked lines are the distribution observed for three replicates. (PDF 11 kb) [file 12864_2016_2921_MOESM9_ESM.pdf]

# CPM

## Paired end reads

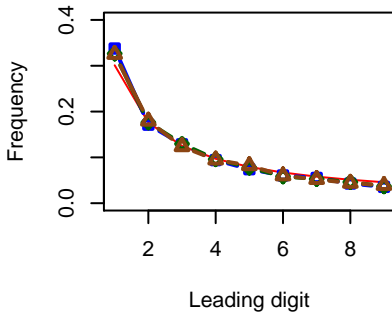

## Single end reads(SE)

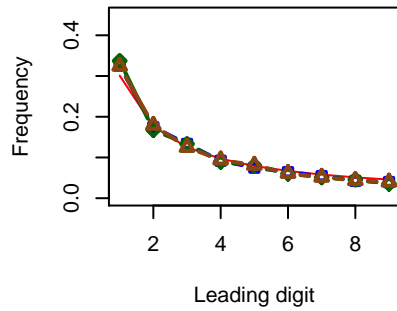

## 25bp(SE)

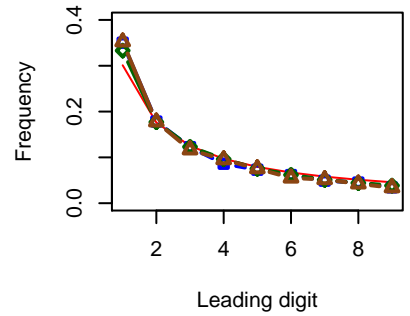

## 50bp(SE)

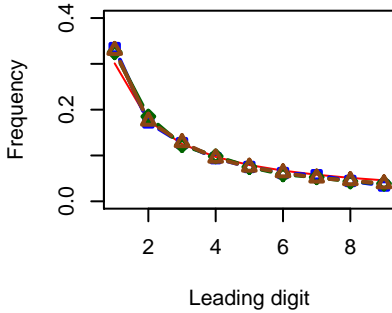

## 30%(SE)

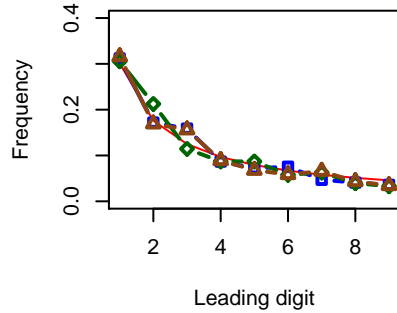

## 50%(SE)

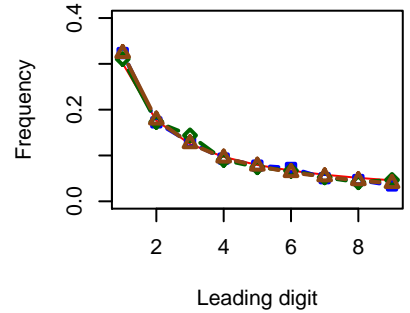

## 80%(SE)

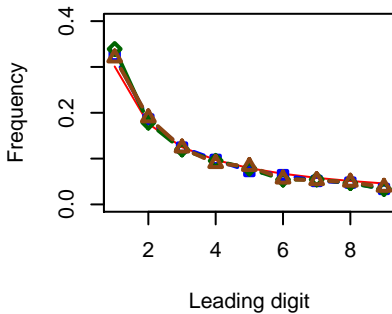

Supplement: Additional file 10: Figure S10. — The effect of different technical parameters on the Benford pattern as calculated based on cell line-derived gene expression data described as CPM values, ignoring very low expressed genes (CPM < 1). If not mentioned otherwise read length was 100 bp and all reads were used in the analysis. Truncated reads (25 and 50 bp) and lower coverage (30, 50 and 80 % out of the total reads) appear in plot titles. The red line indicates the expected Benford distribution, symbol-marked lines are the distribution observed for three replicates. (PDF 11 kb) [file 12864_2016_2921_MOESM10_ESM.pdf]

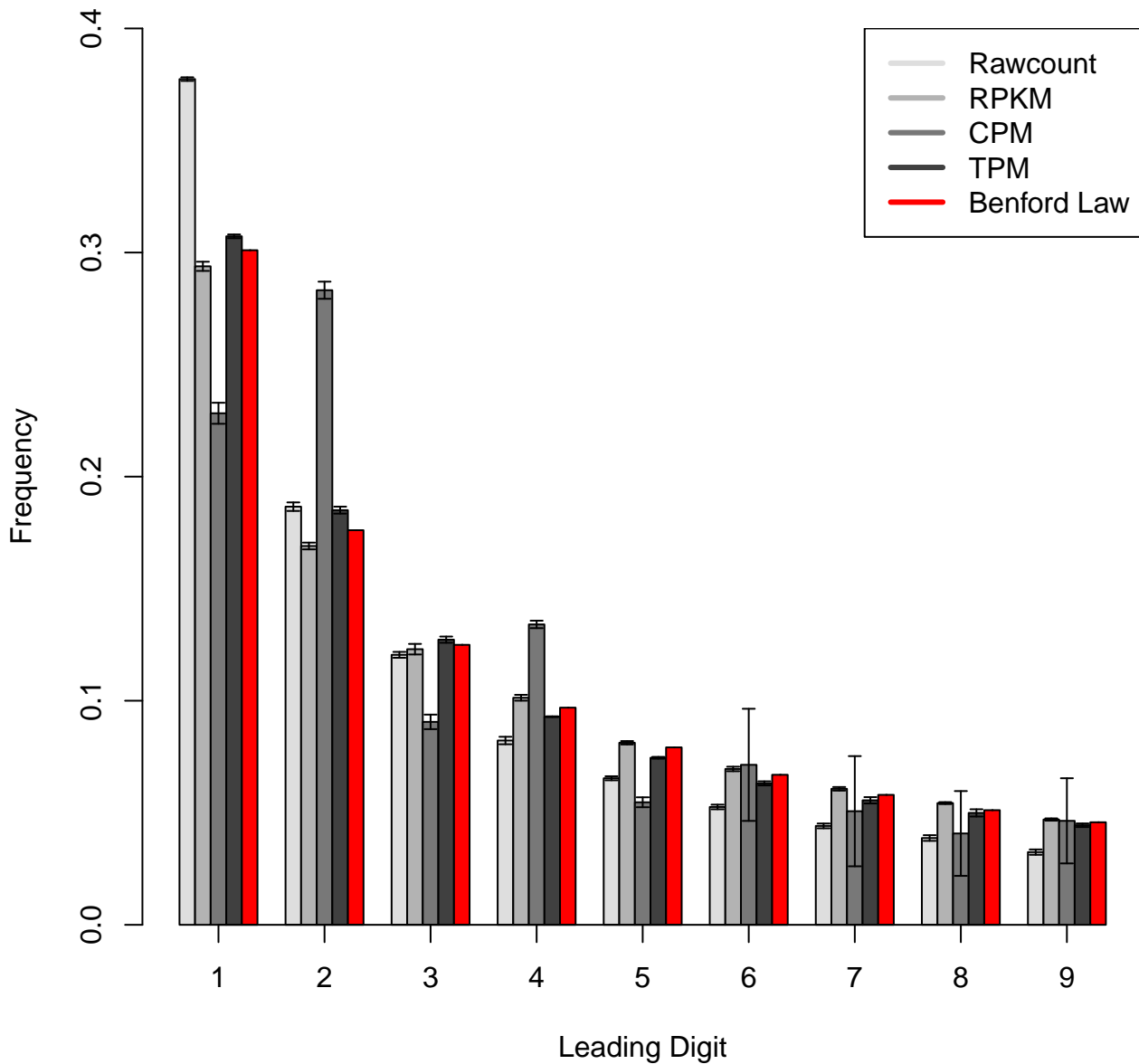

Supplement: Additional file 11: Figure S11. — First digit frequencies of expression data, calculated for different expression metrics. Expression data was calculated based on 100 bp single-end reads of the Universal Human Reference RNA-seq. The mean + SD across three replicates are shown. Black bars represent the expected Benford distribution. (PDF 11 kb) [file 12864_2016_2921_MOESM11_ESM.pdf]

**a**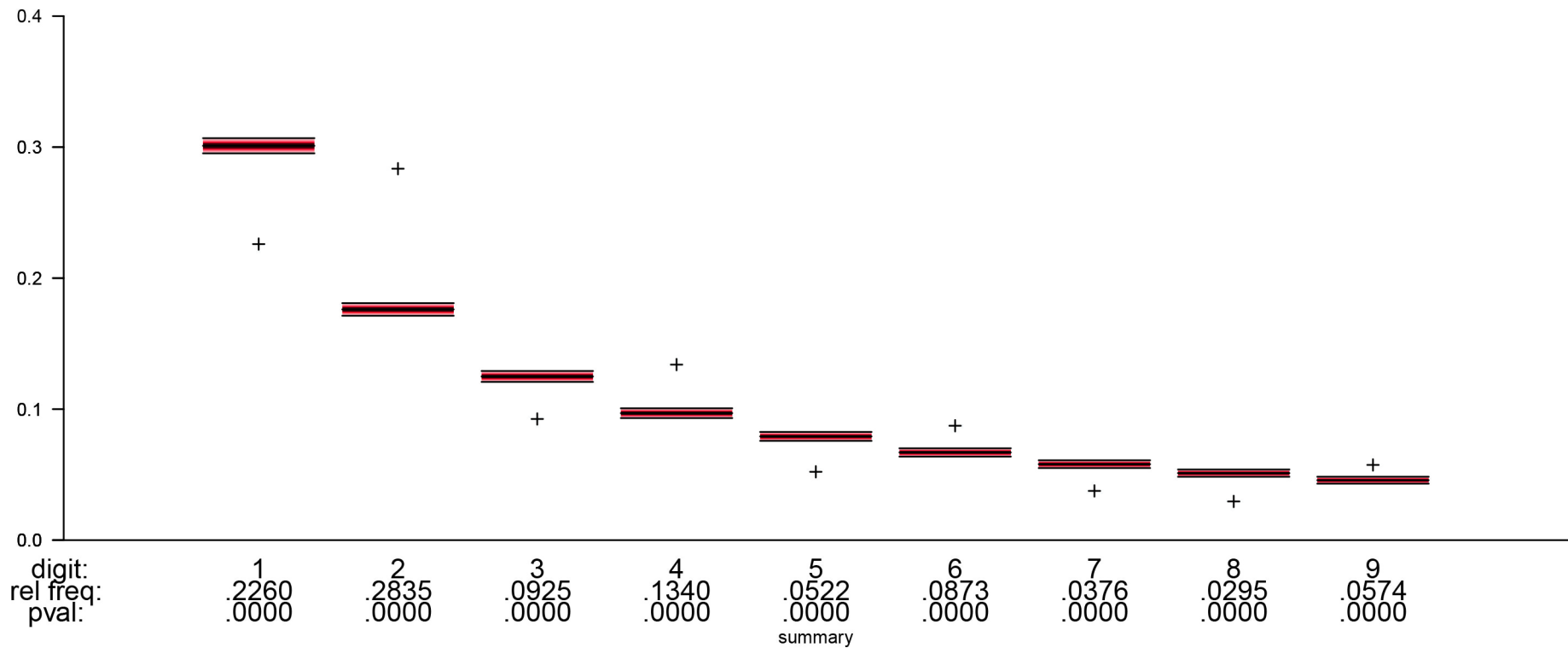**b**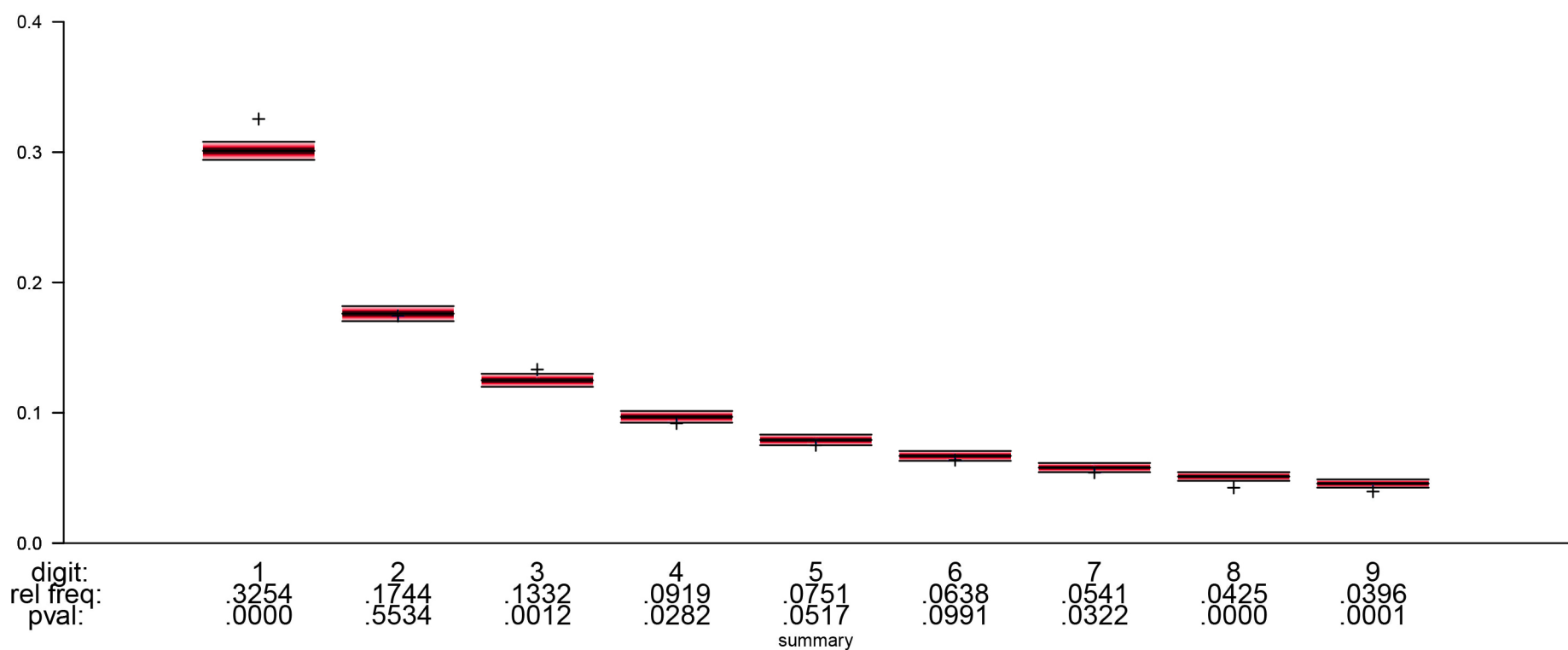**c**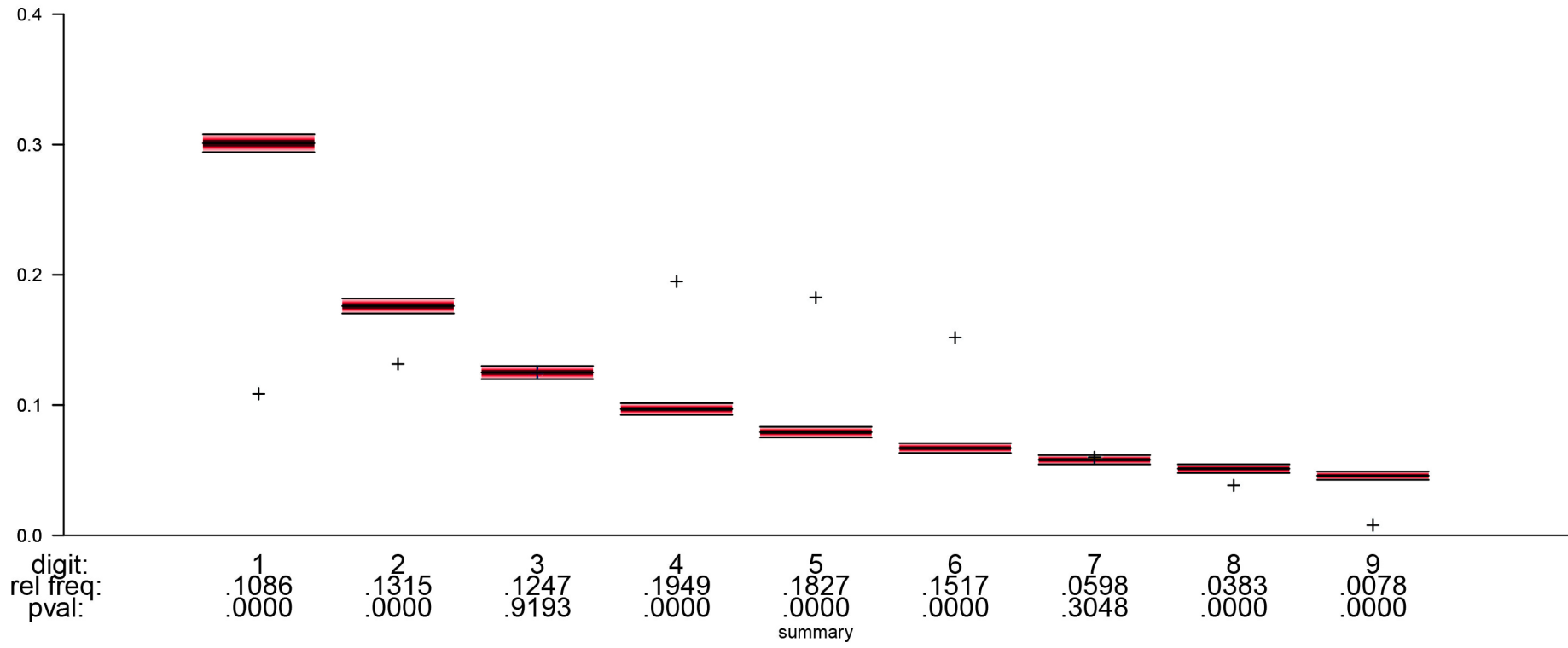

Supplement: Additional file 12: Figure S12. — First digit distributions of the expression counts for a sample dataset (100 bp single-end reads of the universal human reference RNA-seq). First digit frequencies were calculated based on counts per million mapped reads (CPM) for all genes having (a) CPM > 0 (b) CPM > 1 (c) First digit frequencies were calculated based on log 2 of the CPM counts for all genes having CPM > 1. Red lines represent the Benford first digit frequencies together with confidence intervals. Black pluses represent the observed frequencies. Observed relative frequencies and p values are summarized below the plot (see the signifd.analysis command in the BenfordTests package for more details on the calculations). (PDF 1805 kb) [file 12864_2016_2921_MOESM12_ESM.pdf]

# Lung-Tissue

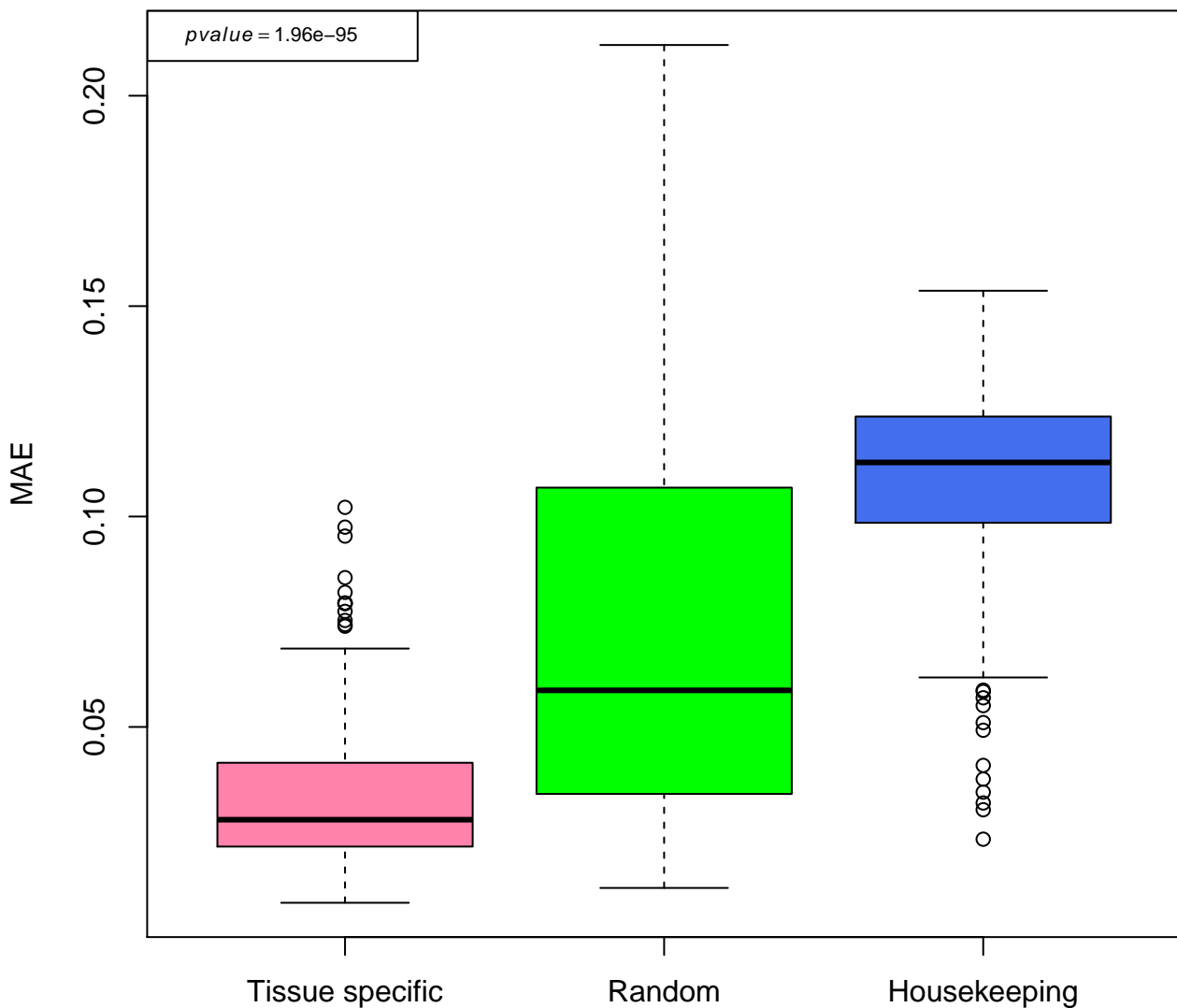

Supplement: Additional file 14: Figure S13. — Expression deviation of different gene sets from the Benford distribution. The MAE (mean absolute error) was calculated across 133 lung tissues for every gene included in the housekeeping, tissue specific and random gene sets (gene-centric mode). A one-sided Mann–Whitney U test was computed to compare between tissue-specific and housekeeping distributions, and the p values are indicated in the plot. (PDF 5 kb) [file 12864_2016_2921_MOESM14_ESM.pdf]

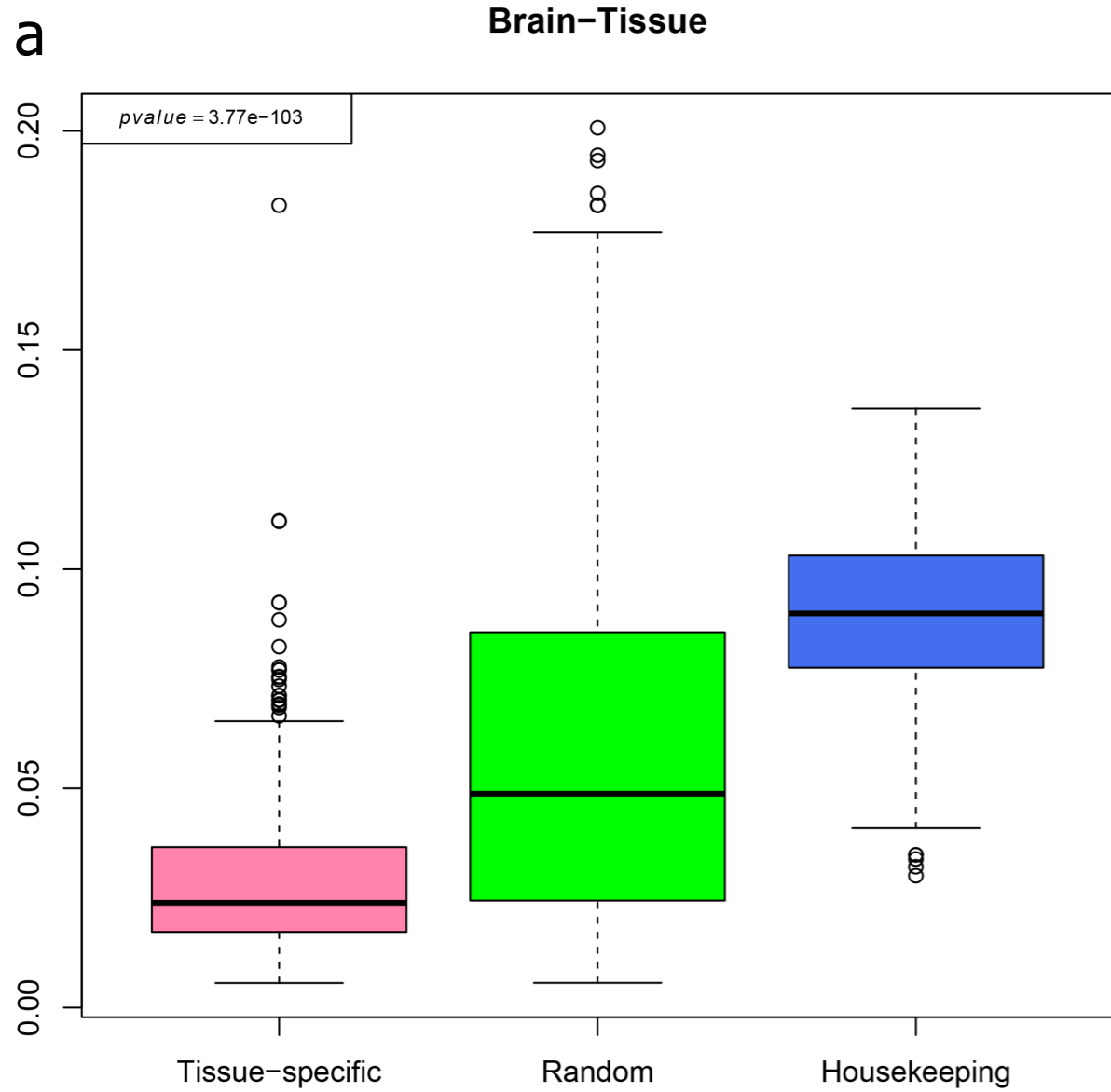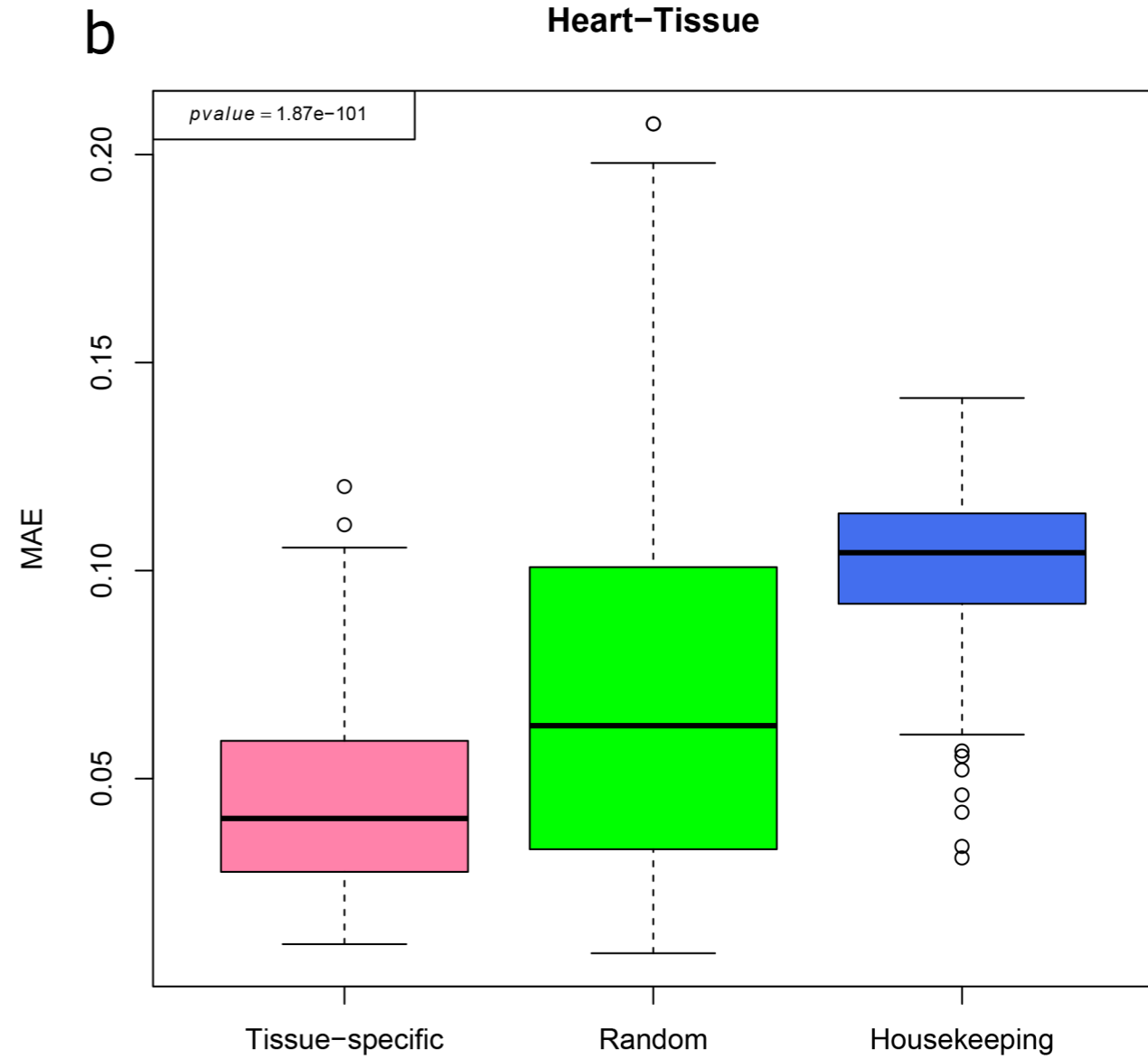

Supplement: Additional file 15: Figure S14 — Expression deviation of different gene sets from the Benford distribution. The MAE (mean absolute error) distribution was calculated across (a) 357 brain tissues and (b) 133 heart tissues, for every gene included in the housekeeping, tissue specific and random gene sets (gene-centric mode). A one-sided Mann–Whitney U test was computed to compare between tissue-specific and housekeeping distributions, and the p values are indicated in the plot. (PDF 707 kb) [file 12864_2016_2921_MOESM15_ESM.pdf]

**Embryo**

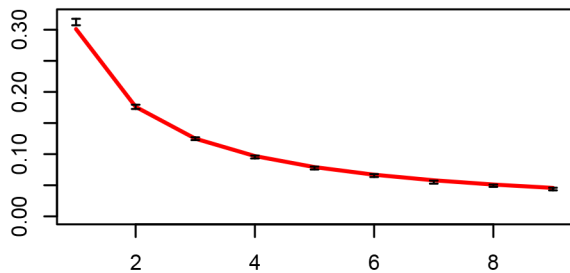

**Larvae**

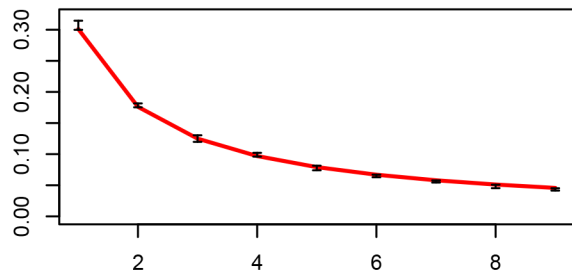

**White pre-pupa**

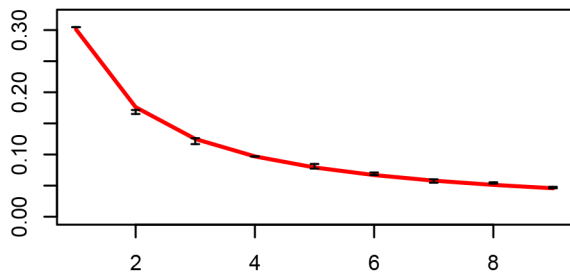

**Adult female**

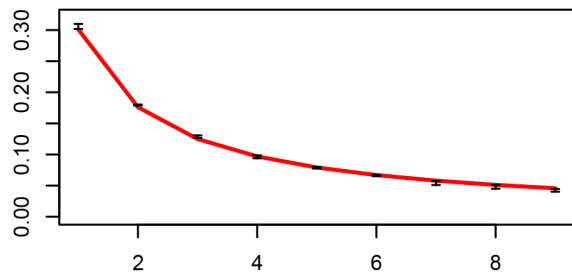

**Adult male**

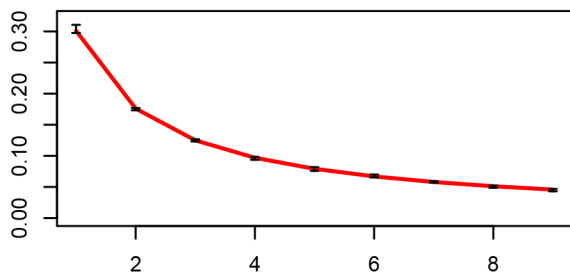

Supplement: Additional file 16: Figure S15. — The proportional frequency of each leading digit as predicted by the Benford distribution (solid line) and observed in Drosophila RNA-seq data at various developmental stages, as calculated for ~700 genes highly expressed in Adult stage compared with other stages (fold change > 16). The mean + SD across replicates (2 to 12 depending on the developmental stage) was plotted. (PDF 410 kb) [file 12864_2016_2921_MOESM16_ESM.pdf]
