# Supplementary material for: Elucidating tissue specific genes using the Benford distribution
Source: BMC Genomics. 2016 Aug 9;17:595. doi: 10.1186/s12864-016-2921-x (PMC4979126; doi:10.1186/s12864-016-2921-x)
Supplement: Additional file 13: Table S1. — The various expression metrics that were used in different analyses. (PDF 112 kb) [file 12864_2016_2921_MOESM13_ESM.pdf]

Supplementary Table 1: The various expression metrics that were used in different analyses

Dataset	Figure	Expression metric used for Benford test
Mouse liver	1	Raw counts
Illumina body map	3	Raw counts
GTEx lung sample	4,9	RPKM
Retina single cell	5,6	CPM
Drosophila Melanogaster Developmental stages	7	Raw counts
ES single cell	8	CPM
